# Gene Flow Risks From Transgenic Herbicide-Tolerant Crops to Their Wild Relatives Can Be Mitigated by Utilizing Alien Chromosomes

**DOI:** 10.3389/fpls.2021.670209

**Published:** 2021-06-11

**Authors:** Xiaoling Song, Jing Yan, Yuchi Zhang, Hewei Li, Aiqin Zheng, Qingling Zhang, Jian Wang, Qing Bian, Zicheng Shao, Yu Wang, Sheng Qiang

**Affiliations:** Weed Research Lab, College of Life Sciences, Nanjing Agricultural University, Nanjing, China

**Keywords:** *Brassica napus*, *Brassica juncea*, generation progeny, chromosome, mixoploids, transgenes, genetic introgression

## Abstract

Integration of a transgene into chromosomes of the C-genomes of oilseed rape (AACC, 2*n* = 38) may affect their gene flow to wild relatives, particularly *Brassica juncea* (AABB, 2*n* = 36). However, no empiric evidence exists in favor of the C-genome as a safer candidate for transformation. In the presence of herbicide selections, the first- to fourth-generation progenies of a *B. juncea* × glyphosate-tolerant oilseed rape cross [EPSPS gene insertion in the A-genome (Roundup Ready, event RT73)] showed more fitness than a *B. juncea* × glufosinate-tolerant oilseed rape cross [PAT gene insertion in the C-genome (Liberty Link, event HCN28)]. Karyotyping and fluorescence *in situ* hybridization–bacterial artificial chromosome (BAC-FISH) analyses showed that crossed progenies from the cultivars with transgenes located on either A- or C- chromosome were mixoploids, and their genomes converged over four generations to 2*n* = 36 (AABB) and 2*n* = 37 (AABB + C), respectively. Chromosome pairing of pollen mother cells was more irregular in the progenies from cultivar whose transgene located on C- than on A-chromosome, and the latter lost their C-genome-specific markers faster. Thus, transgene insertion into the different genomes of *B. napus* affects introgression under herbicide selection. This suggests that gene flow from transgenic crops to wild relatives could be mitigated by breeding transgenic allopolyploid crops, where the transgene is inserted into an alien chromosome.

## Introduction

One of the concerns about growing transgenic herbicide-tolerant (HT) oilseed rape (*Brassica napus*, AACC = 38) is that HT transgenes may spread to their weedy relatives *via* spontaneous hybridization and introgression. If this were to happen, the weeds with HT traits could create new and serious weed control problems (Kling, 1996; Chèvre et al., 1997; Dale et al., 2002; Chapman and Burke, 2006; Rizwan et al., 2019; Clark and Maselko, 2020). Oilseed rape is predominantly self-fertile; however, it can outcross *via* both wind and insects, with average outcrossing rates of ~30% (Beckie et al., 2003). Furthermore, several weedy relatives are generally within the geographical range where it is grown, especially *B. rapa* (AA = 20) in North America and Europe (Allainguillaume et al., 2006; Johannessen et al., 2006) and *B. juncea* (AABB = 36) in Asia (Huangfu et al., 2011; Tsuda et al., 2012; Dong et al., 2018); both of which are highly likely to cross with *B. napus* (Scheffler and Dale, 1994; Bing et al., 1996; Jørgensen et al., 1998; Liu et al., 2010, 2013a,b, 2018; Tsuda et al., 2012).

Introgressive hybridization is the genetic modification of one species by another through hybridization and repeated backcrossing (Anamthawat-Jónsson, 2001). The introgression of a transgene, like any other gene, is a multigenerational process (Mikkelsen et al., 1996). The first step for transgene escape is the spontaneous hybridization between a transgenic crop and a compatible weed or wild relative. Besides the initial crop-weed hybridization, gene transmission during successive generations, the production of fertile and fit offspring through successive backcrosses or selfing, and effective gene recombination between genomes are also important determinants of introgression (Chèvre et al., 1998, 2007; Jenczewski et al., 2003; Jørgensen et al., 2009).

The long-term persistence of a HT trait in subsequent generations of wild or weedy plants is an important factor for introgression. The probability of crop genes transferring to weedy relatives depends on the level of genetic and structural homology between them, as well as the strength for the selection of the transgene in the weedy relative (Jenczewski et al., 2003). The specific integration position of transgenes also affects their transmission frequency (Zhu et al., 2004; Tizaoui and Kchouk, 2012). Theoretical predictions suggest that gene introgression from *B. napus* to its relatives, *B. rapa* or *B. juncea*, should occur more easily when the transgene is originally carried by the A-chromosomes of *B. napus* rather than the C-chromosomes, as the C-chromosomes have no homologous partners during meiosis (Tomiuk et al., 2000). The persistence of HT trait from *B. napus* to *B. rapa* deviated significantly from Mendelian segregation under herbicide selection (Metz et al., 1997). The transgene for glyphosate tolerance was previously observed to be more easily introgressed from *B. napus* into *B. juncea* than the transgene for glufosinate tolerance, due to a lack of stabilized introgression (Song et al., 2010). The most plausible explanation for this^.^ was that the transgene for the glufosinate tolerance was on one of the C-chromosomes, which are lacking in *B. juncea*, but there are currently no reports with empiric evidence to describe such intrinsic mechanisms. This led us to hypothesize that the differences in introgression could be due to differences in the location of transgenes on the A- or C-chromosomes. If the possibility of persistence of HT trait was lower, it could effectively reduce the possibility of transgene introgression from a transgenic crop to a weedy relative under herbicide selection. However, the behavior of transgenes that are inserted into the C-chromosomes and introgressed into wild relatives is currently unknown.

The likelihood of transgenes spreading by introgression from crops into related weedy populations depends in part on the fitness of the first and successive generations of hybrids (Hauser et al., 1998a,b; Gueritaine et al., 2002; Jenczewski et al., 2003; Lu and Snow, 2005; Liu et al., 2010, 2013a,b, 2018). Variations in fitness are expected across the subsequent hybrid generations, due to recombination and selection (Jenczewski et al., 2003). Increased fitness from the F1 or F2 hybrids to backcrossed generations was confirmed between *B. napus* and the first-ranked cross-compatible recipient *B. rapa* (Hauser et al., 1998a,b; Pertl et al., 2002; Ammitzbøll et al., 2005; Allainguillaume et al., 2006). Similarly, the female fertility of *B. napus*–*Raphanus raphanistrum* hybrids increased continuously over successive backcross generations (Chèvre et al., 1997).

Crop- to wild- or weedy-relative gene flow has usually occurred in the context of interspecific hybridizations; i.e., effective gene introgression occurs when there is recombination between genomes of the transgenic crop and its wild or weedy relatives (Jenczewski et al., 2003; Chèvre et al., 2007). Hybrid genomes, consequently, may help to clarify the evolution of hybrid genome structures during successive generations. The chromosome numbers of the F1 hybrids or advanced generations between transgenic oilseed rape and its relatives, the most compatible weed *B. rapa* (Mikkelsen et al., 1996; Pallett et al., 2006) and the more distantly related, *R. raphanistrum*, have previously been studied (Chèvre et al., 1997, 1998, 2007; Gueritaine et al., 2002; Al Mouemar and Darmency, 2004) using root meristems or by estimating nuclear DNA content. Few reports on the chromosome constitutions of hybrids or the advanced generations between transgenic oilseed rape and its relatives are available, except for BC2 plants from transgenic oilseed rape and *R. raphanistrum* (Chèvre et al., 2007). Moreover, the transgene locations in the transgenic oilseed rape remain unclear.

After crop- to wild- or weedy-relative hybridization, some of the crop genomic segments may become established in weedy populations through the selfing of hybrids or through backcrosses to the wild or weedy parents. The likelihood of transgene introgression will not only be determined by the fitness effects from the transgene itself, but also by the crop genes linked to it (Uwimana et al., 2012). Although “domestication” traits are typically considered unlikely to persist in wild populations, some traits may enhance hybrid fitness, especially in stressful environments (Mercer et al., 2007). Through molecular marker assays, the extensive transfer of nuclear as well as plastid DNA from oilseed rape into a self-maintained weedy population of *B. rapa* has been documented in agricultural conditions (Hansen et al., 2001). In addition, the persistence of the C-chromosome regions was identified in backcross progenies between *B. juncea* and *B. napus* (Frello et al., 1995; Tsuda et al., 2012; Guan et al., 2020). Specific alleles of cultivated radish (*Raphanus sativus*) have also been found to persist for 10 years in four different populations of *R. raphanistrum*, and the fecundity of the plants from the experimental populations over this time was found to be similar to that of the wild genotypes (Snow et al., 2010).

After *B. rapa, B. juncea* is the second most cross-compatible recipient of the gene flow from *B. napus* (Scheffler and Dale, 1994). The spontaneous or hand hybridization of conventional and transgenic oilseed rape with *B. juncea* has previously been reported (Scheffler and Dale, 1994; Frello et al., 1995; Bing et al., 1996; Jørgensen et al., 1998; Pu et al., 2005; Song et al., 2007; Liu et al., 2010, 2018; Cao et al., 2014). HT transgenes were transmitted relatively easily to F1 hybrids and retained their activity when transgenic oilseed rape was used as the male or female progenitor. However, the sexual fertility of the F1 hybrid carrying the transgene was low (Song et al., 2007; Liu et al., 2010). The reproduction of F2 and BC1 progenies between herbicide-resistant *B. napus* and wild *B. juncea* was lower than that in *B. napus* under field conditions (Liu et al., 2010). Four successive reciprocal backcrosses between F1 (transgenic HT *B. napus* as pollen donor) or subsequent HT backcross progenies and wild *B. juncea* obtained by hand pollination were used to assess the potential flow of transgenes. The HT first reciprocal backcross progenies produced fewer siliques per plant than the wild *B. juncea* (Song et al., 2010). However, no previous investigations have examined the fitness, the chromosomal compositions of advanced progeny generations of BC1 that developed from transgene oilseed rape, and the effects of transgene placement on the A vs. C genomes for ease of introgression.

We aim to reveal the different mechanisms involved in the introgression of transgenes located on A- and C-chromosomes under herbicide selection by addressing the following three questions: (1) How do chromosomes change across four self-pollination generations of BC1? (2) How does transgene insertion on the chromosomes of the A or C genomes affect the processes of introgression from oilseed rape (AACC) into its wild relative *B. juncea* (AABB)? (3) What is the fate of C- chromosomes of oilseed rape introgressed into *B. juncea* over four generations?

## Materials and Methods

### Plant Materials

Glyphosate-tolerant (Roundup Ready, event RT73) oilseed rape and glufosinate-tolerant (Liberty-Link, event HCN28) oilseed rape (*B. napus* L.; genome, AACC; diploid chromosome number, 2*n* = 38) were provided by Dr. Wenming Zhang, Agriculture and Agri-Food Canada (Ottawa Research and Development Centre). RT73 (carrying *cp4-epsps* and *gox* transgenes that confer glyphosate tolerance) and HCN28 (carrying the *pat* transgene that confers glufosinate tolerance) were produced from spring-type *B. napus* cv. Westar and *B. napus* cv. AC Excel, respectively, by the International Service for the Acquisition of Agri-biotech Applications (http://www.isaaa.org/). The *cp4-epsps* and *gox* transgenes in RT73 are located on chromosomes in the A-genome, while the *pat* transgene in HCN28 is located on a chromosome in the C-genome (Supplementary Material 1). Wild *B. juncea* seedlings were identified in a winter wheat field in Jiangpu, Nanjing City, China, according to the SSR markers (Sun et al., 2018). The collected seedlings were transplanted and self-pollinated as described previously by Song et al. (2010).

The study was conducted from 2010 to 2017. F1 interspecific hybrids were obtained by hand pollination, using at least 20 wild *B. juncea* plants (maternal) crossed with the pollen of 20 glyphosate-tolerant oilseed rape (paternal) and 20 glufosinate-tolerant oilseed rape (paternal) individuals, as described previously by Song et al. (2010). Specifically, half of the flowers of each single maternal wild *B. juncea* plant were pollinated using a glyphosate-tolerant oilseed rape and the other half with a glufosinate-tolerant oilseed rape.

F1(R) and F1(L) were used to identify the F1 generations that were obtained using the glyphosate- and glufosinate-tolerant oilseed rape as the paternal plants, respectively. The mature seeds of the F1(R) and F1(L), produced by the different maternal plants, were harvested separately and stored in dark conditions in a refrigerator at 4°C, until further use. Seeds were planted, and emerged seedlings were sprayed with glyphosate or glufosinate at the fourth- to fifth-leaf stage, as described in the “Methods” section, and survivors, including F1R or F1L (R means glyphosate-tolerant plants, L means glufosinate-tolerant plants), were used for backcrossing.

**Figure 1:**
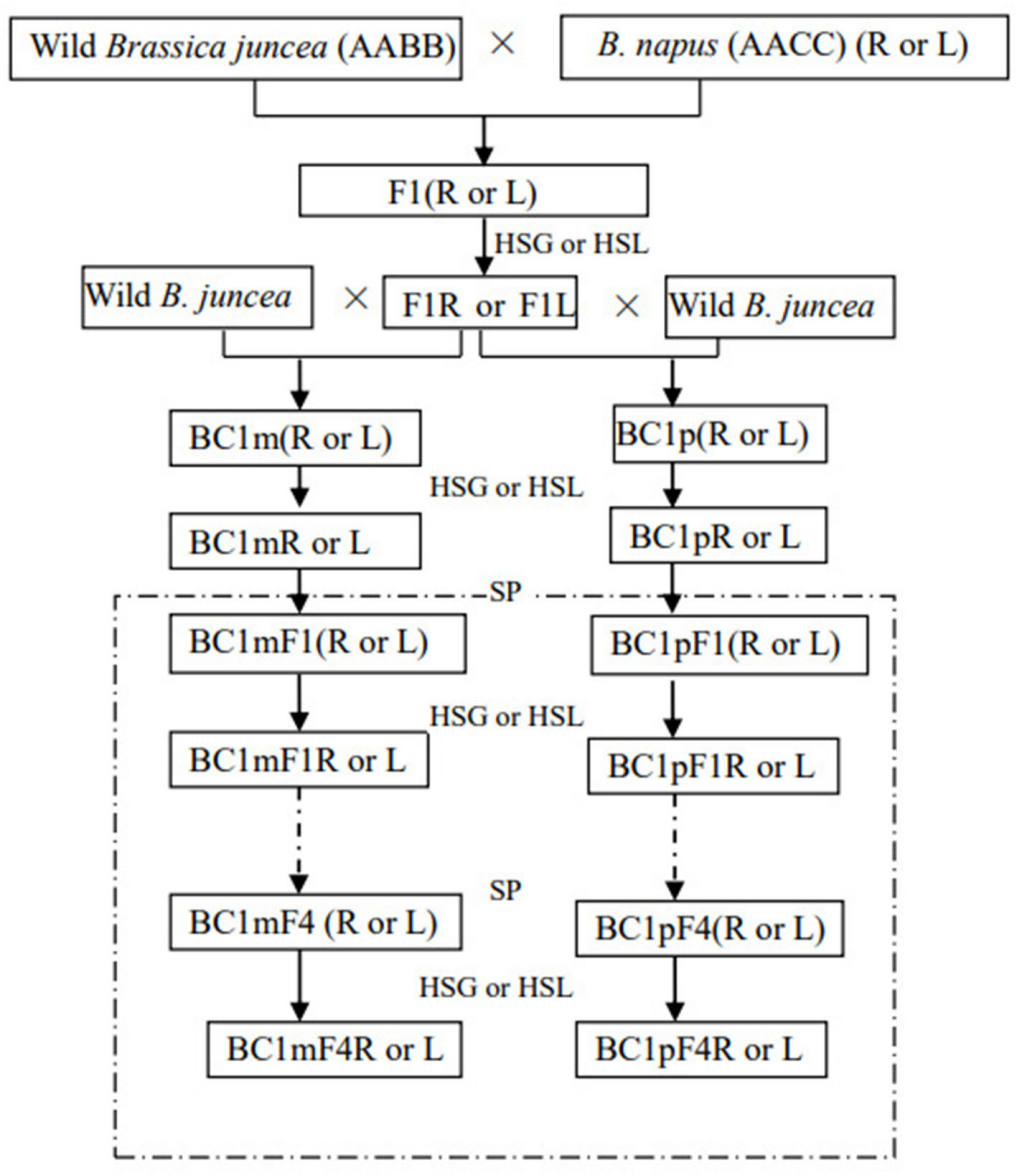
Crossing scheme for the hybridization and backcrossing of wild *Brassica juncea* and transgenic herbicide-tolerant oilseed rape, and self-pollination of the first backcross generation (BC1). Combinations involved in this study are indicated as maternal plants × paternal plants. m and p indicate the backcross generations obtained with wild *Brassica juncea* as the maternal and paternal plants, respectively. (R) and (L) indicate the F1, the first backcross generation, and its progeny obtained by using the transgenic glyphosate- or glufosinate-tolerant oilseed rape as the paternal plants, respectively. R means glyphosate-tolerant transgenic oilseed rape, F1, and backcross generation. L means glufosinate-tolerant transgenic oilseed rape, F1, and backcross generation. BC1F1 to BC1F4 are the first- to fourth-generation progenies of the first backcross generation (BC1). SP indicates self-pollination, and HSG and HSL indicate herbicide selection by glyphosate or glufosinate, respectively.

A schematic presentation of the crosses is provided in Figure 1. To avoid the effects of polymorphism from the wild *B. juncea*, separate flowers of a single maternal plant of wild *B. juncea* were pollinated with either F1R or F1L, and pollen from a single paternal plant of wild *B. juncea* was used to pollinate F1R and F1L. More than 100 flowers were crossed per maternal plant, and at least fifteen mother plants were pollinated in each backcross combination, which were as follows: wild *B. juncea* × F1R; F1R × wild *B. juncea*; wild *B. juncea* × F1L; and F1L × wild *B. junce*a. Seeds from the same mother plants of each backcross combination were defined as being in the same plant lineage. BC1 seeds obtained from the different plant lineages were harvested separately and stored in the dark at 4°C, until further use. At least 500 BC1 seeds (50 from each of the 10 plant lineages) were sown. The plants of BC1 that survived after glyphosate or glufosinate treatments were cultured until flowering. The inflorescences of these plants were bagged before anthesis and allowed to self-pollinate to produce the first-generation progeny of BC1, including BC1mF1(R), BC1pF1(R), BC1mF1(L), and BC1pF1(L). The first-generation progeny that survived after glyphosate or glufosinate treatments were denoted BC1mF1R, BC1pF1R, BC1mF1L, and BC1pF1L, and the process was repeated for the second-, third-, and fourth-generation progeny.

## Methods

### Selection for Tolerance to Herbicide and Fitness-Associated Traits in the Backcross Progeny

For the four different backcross combinations, the 10 mother plants in each generation that displayed the highest seed fertility for each lineage were chosen. At least 50 filled seeds from each of the 10 mother plants were selected randomly, and each seed was sown directly into individual plastic pots (6 cm in diameter) containing growth media (a mixture of garden soil and peat at 1:1 (v/v) ratio) for the duration of the investigation. Plants were grown as described previously by Song et al. (2010). Emergence percentage was calculated for each BC1 progeny type 14 days after sowing using 50 pots randomly selected and marked at the sowing day for each lineage, and regarded as one replicate; there were 10 replicates for each progeny type.

Once the plants reached the fourth- to fifth-leaf stage, they were sprayed with 41% glyphosate isopropylammonium AS (Roundup Ultra; Monsanto, St. Louis, MO, USA) at 1,037 g a.i. ha^−1^ or 18% glufosinate SL (Basta, Bayer CropScience, Germany) at 700 g a.i. ha^−1^. The plants were treated by the use of the same procedure as described by Song et al. (2010). After twice herbicide applications, dead plants were counted and mortality rates were determined.

Five plants from each plant lineage that survived the second application of herbicides, totaling fifty plants of each type of BC1 progeny, were randomly selected for PCR testing. A set of primer pairs for PCR analysis were designed using the sequence of the *cp4-epsps* gene in the transgenic oilseed rape, with Primer 6.0 Software, which are as follows:Primer R1: 5′ AAGGCATTCATTCCCATTTG 3′, Primer R2: 5′ TAACATCTTCACCTTCCAAAAG 3′. Another set of primers was similarly designed using the sequence of the *pat* gene, which are as follows: Primer L1: 5′AGGACAGAGCCACAAACACCAC 3′, Primer L2: 5′ACCAACATCATGCCATCCACCA 3′.

Each PCR involved a 25 μL of reaction system: 2.5 μL 10 × Ex Taq buffer, 2 μL MgCl_2_ (25 mmol/L), 2 μL dNTP mixture (each 2.5 mmol/L), 1 μL forward primer and 1 μL reverse primer (10 μmol/L), 0.3 μL TaKaRa Ex Taq DNA polymerase (5 U/μL) [Treasure Biological Engineering (Dalian) co., LTD], 10 ng of genomic DNA and 14.2 μL ddH2O. PCR amplification was performed on a Whatman Biometra TGRADIENT Thermocycler at 94°C for 5 min for the initial denaturation, 15 cycles with 94°C denaturation, 35 cycles of 94°C for 0.5 min, annealing at 55°C for 0.5 min for the *cp4-epsps* and at 58°C for 0.5 min for pat, 72°C elongation for 1 min, and a final extension at 72°C for 10 min. Amplified DNA products were separated on 1% agarose gels at 100 V for 30 min, stained with ethidium bromide, and visualized under UV light.

Uniformly sized plants from the first, second, third, and fourth progenies of BC1 (10 plants from each plant lineage, 100 plants in total) were selected from the plants that survived the glyphosate or glufosinate spray treatments, and transplanted individually into pots (25 cm in diameter) containing the same growth media as described previously. They were grown in greenhouse conditions, exposed to natural light from the date of transplanting to bolting (from the end of November to the end of February of the next year), and from bolting to harvesting (from March to May). Adjacent pots were separated by 10 cm. Pots were laid out in a completely randomized design, and no sexually compatible crucifer species was present for a 500 m radius around the experiment. Inflorescences were bagged before anthesis to allow for self-pollination. Sixty wild *B. juncea* plants (a single replicate consisted of 10 plants from each mother; there were six replicates in total) were used as the controls. Plants were harvested at maturity. Fitness-associated traits were evaluated as described in Supplementary Table 1.

### Chromosome Counting and Constitution

#### Chromosome Counting

The chromosome numbers of the progeny were determined from the ovary cells of the young flower buds according to Li et al. (1995). The treated ovary cells were observed using a light microscope (ZEISS, imager, M2). Seven to ten plants were selected from the first to fourth progeny, and at least 30 cells were analyzed from each plant. When 10 plants were available, we assessed one from each lineage; in some cases, only seven plants were selected, in which case the lineages were selected at random.

#### Chromosome Constitution

In the fourth-generation progeny, three representative plants in BC1mF4R and BC1pF4R, which had the highest proportion of 36 chromosomes, were chosen, and the cells with 36 chromosomes were assessed for chromosome constitution. For BC1mF4L and BC1pF4L, five and seven plants, which had the highest proportion of 37 chromosomes, were chosen, and the cells with 37 chromosomes were assessed for their chromosome constitutions. The following probes were used for combined fluorescence *in situ* hybridization–bacterial artificial chromosome (BAC-FISH), in accordance with Cui et al. (2012).

(1) A 329-bp subfragment of pBNBH35 from *B. nigra* (total genomic DNA of *B. nigra* [BB, 2*n* = 16] provided by Li ZY, Huazhong Agricultural University) bound specifically to the B-genome and not to the A- or C-genomes of *B. napus*, according to the method by Schelfhout et al. (2004). The 329-bp subfragment was labeled with digoxigenin-11-dUTP (Roche, Basel, Switzerland) by random priming using the BioPrime Array CGH Genomic Labeling System kit according to the manufacturer's protocol (Invitrogen, Life Technologies).

(2) The plasmid DNA of the BAC BoB014O06 (also provided by Li ZY) was labeled with biotin-11-dCTP by random priming using the Bio-Prime DNA Labeling System Kit (Invitrogen, Life Technologies) in accordance with the manufacturer's instructions. BAC-FISH was conducted according to Cui et al. (2012). All images were captured with a charge-coupled device camera attached to a fluorescence microscope (ZEISS, imager. M2) with a fluorescence microscope light source (X-cite series 120). Images were processed by Adobe Photoshop CC to adjust the contrast and brightness.

### Meiosis Study

Three representative plants from the BC1mF4R and BC1pF4R generation, which had the highest proportion of 36 chromosomes, and three from the BC1mF4L and BC1pF4L generation, which had the highest proportion of 37 chromosomes, were selected for analysis. For each plant, at least 150 cells cumulatively from the four anthers of four buds were observed for each splitting phase.

To observe the pollen mother cells (PMC) for the meiotic analysis, buds from the terminal inflorescence were fixed immediately after collection in fresh Carnoy's solution for 24 h and then stored at −20°C. The anthers were treated according to Li et al. (1995), and then the treated anthers were observed using a light microscope (ZEISS, imager, M2).

The meiotic index (mi) was defined as the percentage of normal tetrads recorded. Normal tetrads were those with four equal-sized cells. Meiotic indices (mi) were calculated according to the method of Sapra and Heyne (1973) and the formula: mi = (number of normal tetrads/total of tetrads) ×100. Stable inheritance was indicated by a value of 90–100 mi (An et al., 2015).

### Persistence of C Genome Chromosome (C-Chromosome) Regions of *B. napu*s in the Progenies of BC1

For each backcross generation, four seedlings of each plant lineage were chosen randomly. This totaled 40 plants from 10 plant lineages were used to test the persistence of the C-chromosome regions.

#### DNA Isolation and Marker Surveys

DNA was extracted from the leaf tissues of seedlings following the modified CTAB protocol described by Doyle (1991). Purified DNA samples from five independent plants of *B. rapa* (AA), *B. nigra* (BB), *B. napus* (AACC), and *B. juncea* (AABB) were surveyed for 83 SSR markers located on linkage groups N11–N19 (Tsuda et al., 2012). Sixteen markers that were specific to the C-chromosome and were found in *B. napus* but were completely absent from *B. rapa, B. nigra*, and *B. juncea*, were then tested in 40 individual plants of the BC1 progenies (Supplementary Table 2).

Each PCR involved a 10 μL of reaction system: 4.5 μl BU-Taq 2 × master PCR mix (Tianwei of Nanjing Biological Technology Co., Ltd.), 0.25 μl forward primer (8 μM), 0.25 μl reverse primer (8 μM), 10 ng of genomic DNA and 4.0 μl ddH2O. PCR amplification was performed on a Whatman Biometra TGRADIENT Thermocycler at 94°C for 5 min for the initial denaturation, 15 cycles with 94°C denaturation, 60°C annealing for 1 min, 72°C elongation for 1 min, with a 0.7°C decrease in the annealing temperature at each cycle; then 23 cycles with 94°C for 0.5 min, annealing at 55°C for 0.5 min, 72°C elongation for 1 min; then a final elongation step for 10 min at 72°C. The amplified DNA products were separated on a 6% polyacrylamide gel with electrophoresis, at 120 V for 2–3 h, stained with 0.1% AgNO3 in 1 × TBE buffer, and visualized under an ultraviolet illuminator.

The frequencies of the detected markers that were specific to the C-chromosome in the different progeny of the BC1 were calculated as the average of the number of each marker detected/number of tested plants.

### Data Analysis

BC1F1-BC1F4 segregation data for the glyphosate or glufosinate tolerance was tested for goodness of fit with the different genetic ratios using chi-square analyses. Separation of the means of each measured variable was performed following the method described by Song et al. (2011), using SPSS II 20.0 software. The means of each trait (including the vegetative and reproductive stages) were separated using Duncan's multiple range test. To estimate relative fitness, the fitness-associated traits of *B. juncea* were defined as “1,” and every other type was assigned a fitness value based on its ratio to the value assigned to progenies of the transgenic oilseed rape and wild *B. juncea*. The composite fitness was the mean of eight relative fitness estimates from the vegetative to mature stages, including plant height, stem diameter, plant rosette diameter, pollen viability, dry aboveground biomass per plant, silique number per plant, silique length, and seeds per silique. Duncan's multiple range test was also used to analyze the differences in composite fitness.

## Results

### Seedling Emergence, Herbicide Tolerance, and Fitness-Associated Traits of the Progeny of the First Backcross Generation

#### Seedling Emergence

**Figure 2:**
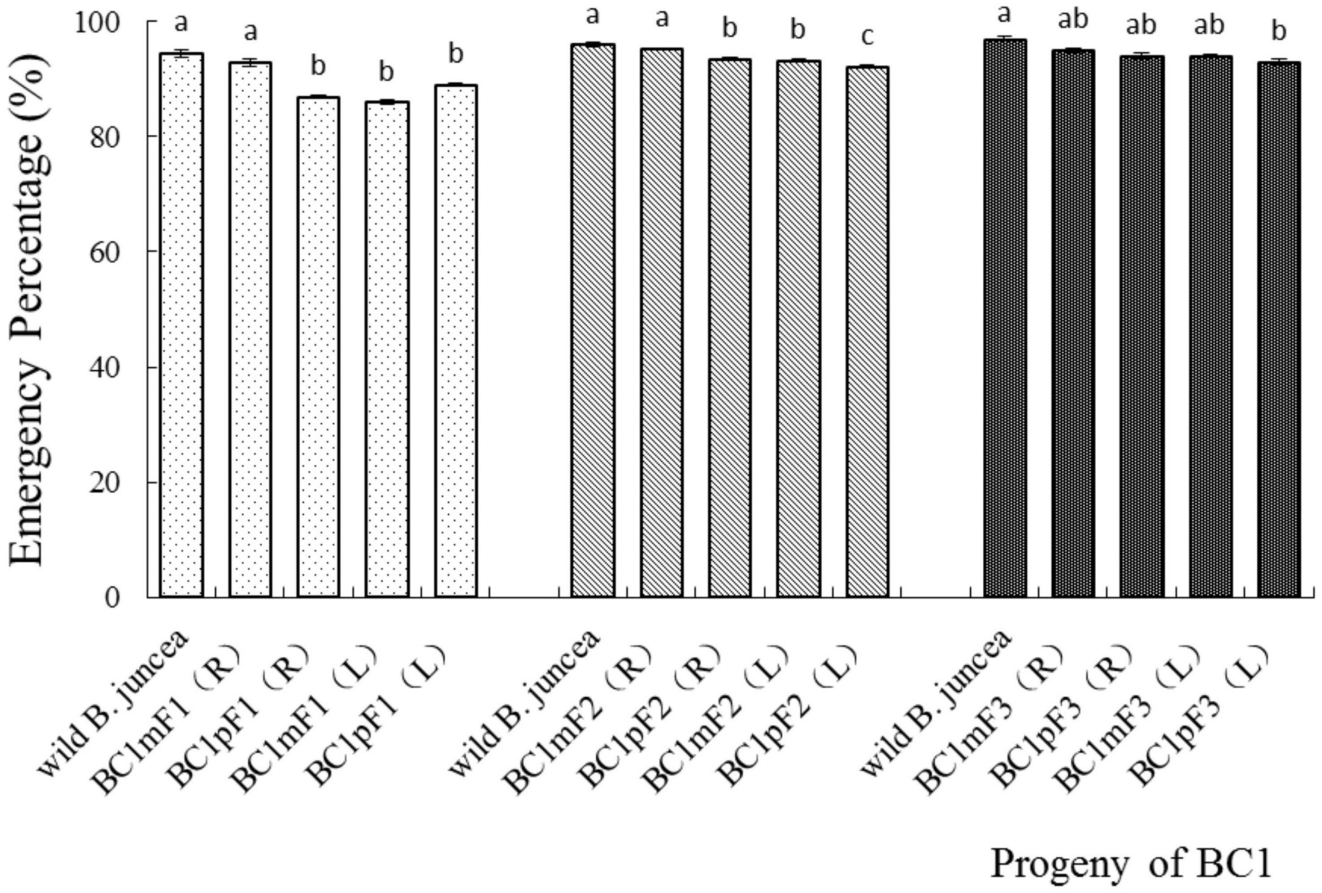
Emergence percentages of the first- to the third-generation progenies of BC1. Data are shown as the mean ± SE (*n* = 10). Different letters in the different progenies of the same generation indicate significant differences using Duncan's multiple range test, *P* < 0.05. BC1mF1(R) to BC1mF4(R) and BC1pF1(R) to BC1pF4(R) are the first- to fourth-generation progenies of the first backcross generation (BC1) obtained from wild *Brassica juncea* × F1R or F1R × wild *B. juncea*, respectively. BC1mF1(L) to BC1mF4(L) and BC1pF1(L) to BC1pF4(L) are the first- to fourth-generation progenies of the first backcross generation (BC1) obtained from wild *Brassica juncea* × F1L or F1L × wild *B. juncea*, respectively. F1R and F1L indicate the glyphosate- or glufosinate-tolerant F1 hybrids from wild *B. juncea* × glyphosate- or glufosinate-tolerant transgenic oilseed rape. Progenitors in front of the × are always the maternal plants, and the progenitors after the × are always the paternal plants.

In the first-generation progeny, the emergence percentages for the BC1mF1(R) and wild *B. juncea* were similar, as both were above 90%. The other three progenies, BC1pF1(R), BC1mF1(L), and BC1pF1(L), had similar emergence percentages, among 86 to 89%, but were significantly lower than those of the wild *B. juncea* and BC1mF1(R). Although all the second-generation progeny had a >92% emergence, BC1pF2(R), BC1mF1(L), and BC1pF1(L), had lower percentages of emergence than the BC1mF2(R) and wild *B. juncea*. In the third-generation progeny, BC1pF3L had 93% emergence, which was lower than that of the wild *B. juncea* (Figure 2). The emergence percentage for all BC1F4 was more than 92%, similar to that of the wild *B. juncea* (data not shown).

#### Herbicide Tolerance Segregation in Progeny

**Figure 3:**
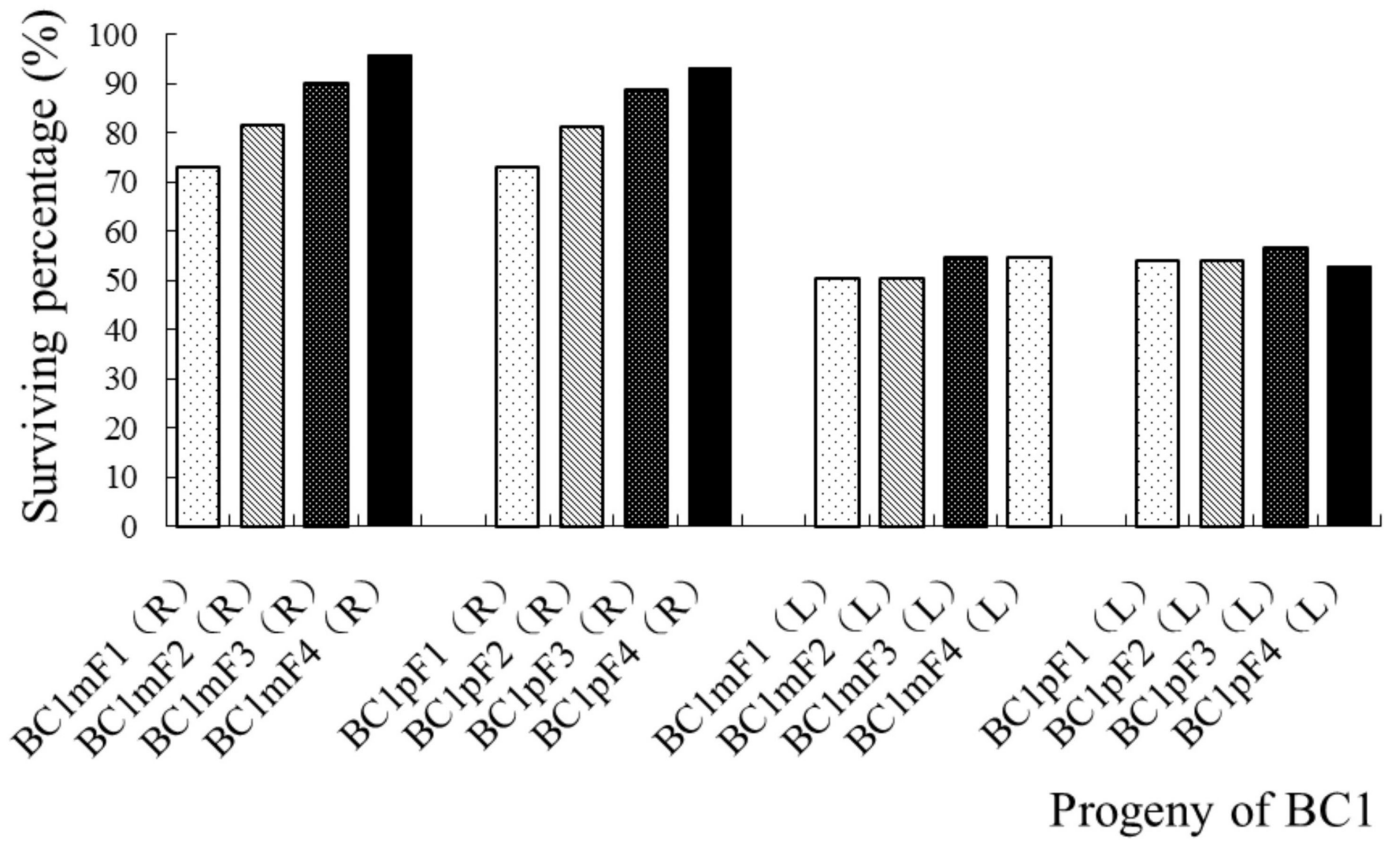
Surviving percentage of the first- to fourth-generation progenies of BC1 after spraying with glyphosate or glufosinate. BC1mF1(R) to BC1mF4(R) and BC1pF1(R) to BC1pF4(R) are the first- to fourth-generation progenies of the first backcross generation (BC1) obtained from wild *Brassica juncea* × F1R or F1R × wild *B. juncea*, respectively. BC1mF1(L) to BC1mF4(L) and BC1pF1(L) to BC1pF4(L) are the first- to fourth-generation progenies of the first backcross generation (BC1) obtained from wild *Brassica juncea* × F1L or F1L × wild *B. juncea*, respectively. F1R and F1L indicate glyphosate- or glufosinate-tolerant F1 hybrids from wild *B. juncea* × glyphosate- or glufosinate-tolerant transgenic oilseed rape. Progenitors in front of the × are always maternal plants, and progenitors after the × are always paternal plants. The dosage of glyphosate used was 1,037 g (a.i.) ha^−1^ and that of glufosinate was 700 g (a.i.) ha^−1^.

None of the wild *B. juncea* control plants survived the herbicide spray treatments, but all the transgenic oilseed rape plants did. The surviving number and percentage of the first to fourth progenies of the BC1, after the glyphosate spray treatments, are provided in Figure 3, Supplementary Table 3-1. The segregation of glyphosate tolerance, which transgene located on A-chromosome, followed the 3:1, 5:1, 9:1, and 17:1 normal Mendelian segregation ratios, which equated to ~73, 81, 90, and 93% of plants surviving the glyphosate spray, respectively. The segregations of glufosinate tolerance, which transgene located on C-chromosome, were highly biased when compared with normal Mendelian segregations, and ~50% plants survived the glufosinate spray in the first to fourth progenies of the BC1 (Figure 3, Supplementary Table 3-2). The results of the tolerance gene PCR amplifications showed that the *cp4-epsps* (549 bp) and *pat* gene (390 bp) fragments from the transgenic oilseed rape were highly conserved in the first to fourth progeny of the BC1 (Supplementary Figures 1-1,1-2).

#### Fitness-Associated Traits and Composite Fitness

**Figure 4:**
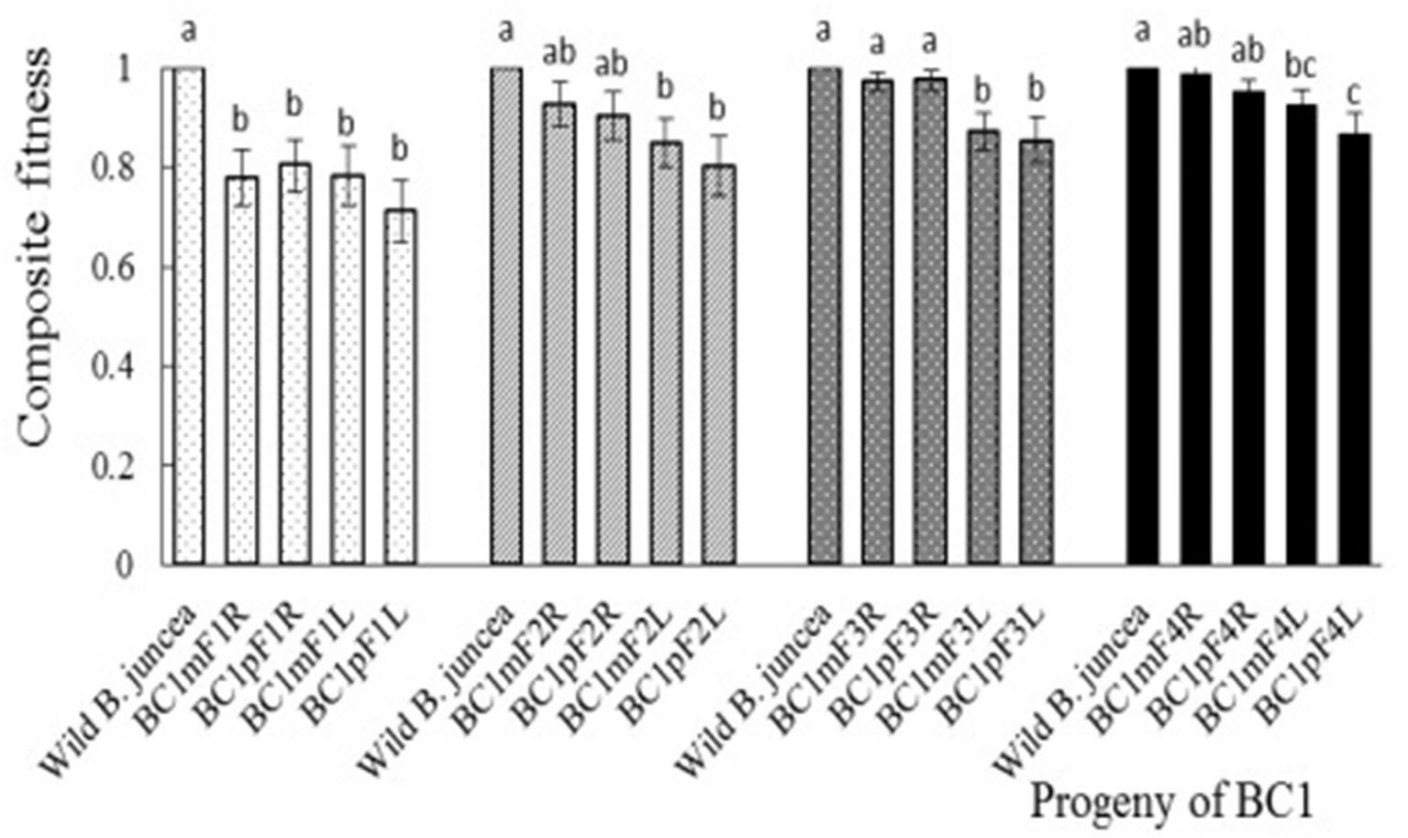
Composite fitness of the first- to fourth-generation progenies of BC1. To estimate relative fitness, the agronomic traits of *Brassica juncea* were defined as “1.” Data were shown as the mean ± SE (*n* = 8). Different letters in the different progenies of the same generation indicate significant differences using Duncan's multiple range test, *P* < 0.05. BC1mF1R to BC1mF4R and BC1pF1R to BC1pF4R are the glyphosate-tolerant first- to fourth-generation progenies of the first backcross generation (BC1), obtained from wild *Brassica juncea* × F1R or F1R × wild *B. juncea*, respectively. BC1mF1L to BC1mF4L and BC1pF1L to BC1pF4L are the glufosinate-tolerant first- to fourth-generation progenies of the first backcross generation (BC1) obtained from wild *Brassica juncea* × F1L or F1L × wild *B. juncea*, respectively. F1R and F1L indicate glyphosate- or glufosinate-tolerant F1 hybrids obtained from wild *B. juncea* × glyphosate- or glufosinate-tolerant transgenic oilseed rape. Progenitors in front of the × are always the maternal plants, and the progenitors after the × are always the paternal plants.

All plants from the first to fourth progeny of the BC1 grew and bloomed vigorously, and no plants died from transplantation to harvest. Nevertheless, the first-generation progeny of BC1 from *B. napus*, whose transgene located on A- or C-chromosome, had reduced fitness, relative to the wild *B. juncea*, for almost all fitness components and for composite fitness. Although plant height and seeds per silique of all the second-generation progenies were significantly lower than those of the wild *B. juncea*, the composite fitness of the glyphosate-tolerant second-generation progenies from *B. napus* with transgene on A-chromosome was similar, while the glufosinate-tolerant second-generation progenies from *B. napus* with transgene on C-chromosome remained lower. In the third- and fourth-generation progeny, the glyphosate-tolerant progeny, compared with the *B. juncea*, had a lower stature, but were compensated by their similar stem and rosette diameters, pollen viability, dry aboveground biomass per plant, seeds per siliques, and silique number per plant. The glufosinate-tolerant progeny remained less fit than the wild *B. juncea*, although increased female fertility was observed in the first- to fourth-generation progeny. Therefore, the fitness-associated traits and composite fitness were restored with the self-pollination of BC1 (Figure 4, Supplementary Figures 2-1–2-8).

### Chromosome Number, Constitution of Ovary Cells, and Chromosome Behavior During Meiosis

#### Chromosome Number

Variable chromosome numbers were recorded in the somatic tissues (ovary cells) of all the first to fourth HT progeny of the BC1. All the first to fourth progeny of the BC1 from *B. napus* whose tolerant transgene located either on A- or on C-chromosome were mixoploids, with 2n > 45 being the maximal chromosome number. This maximum number was a rare occurrence, and thus, those results were not considered further in the analysis. The variation in the chromosome numbers decreased progressively (BC1F4 < BC1F3 < BC1F2 < BC1F1) with each self-pollination generation. As the generations advanced, the percentage of cells with 36 or 37 chromosomes increased for the progenies from *B. napus* whose transgene is located on A- and C-chromosome, respectively, as described below.

**Figure 5:**
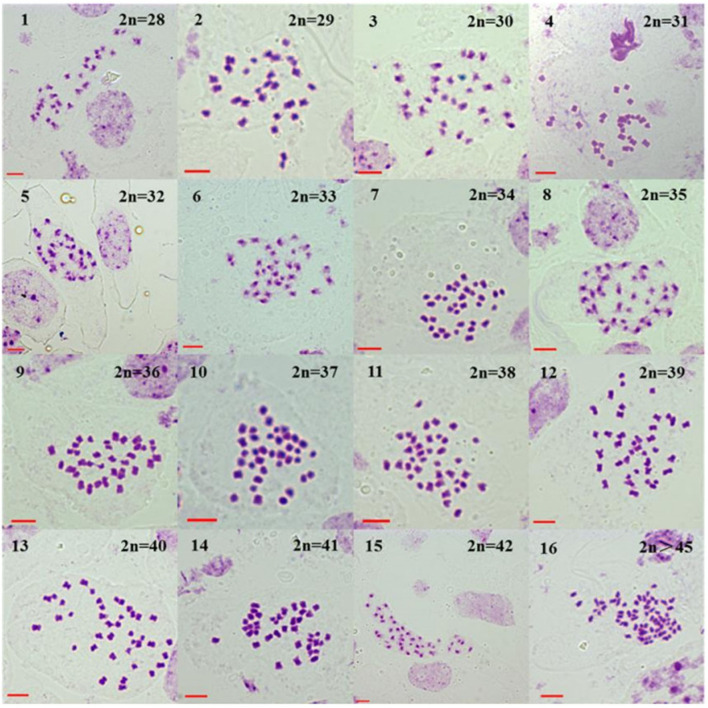
Somatic chromosome numbers of the glyphosate-tolerant first-generation progeny of BC1 (BC1mF1R). BC1mF1R indicates the glyphosate-tolerant first-generation progenies of the first backcross generation (BC1) obtained from wild *Brassica juncea* × F1R. F1R indicates glyphosate-tolerant F1 hybrids obtained from wild *B. juncea* × glyphosate-tolerant transgenic oilseed rape. Progenitors in front of the × are always the maternal plants, and the progenitors after the × are always the paternal plants. All bars: 5 μm.

For the four-generation progenies from *B. napus* whose transgene is located on A-chromosome, the average chromosome numbers are shown in Table 1, and the different chromosome numbers are shown in Figure 5 (BC1mF1R, for example). The first-generation progeny had 27–44 chromosomes (Supplementary Tables 4-1,4-2), the second-generation had 26–40 (Supplementary Tables 4-3,4-4), the third-generation had 26–38 (Supplementary Tables 4-5,4-6), and the fourth-generation had 26–44 (Supplementary Tables 4-7,4-8). From the second- to fourth-generation progeny, plants displayed very particular chromosome number distributions, with a large proportion (> 50 % of cells in most cases) having 2*n* = 36 chromosomes.

**Table 1:** Average percentage of ovary cells with different chromosomes in the glyphosate-tolerance first- to fourth-generation progenies of BC1.

**Progeny**	**RCN**	**Chromosome number**											
		**<35**	**35**	**36**	**37**	**38**	**39**	**40**	**41**	**42**	**43**	**44**	**>45**
BC1mF1R	28–42	8.04	4.14	16.79	10.94	18.02	13.95	16.35	6.03	0.82	0	0	5.52
BC1pF1R	27–44	8.03	12.88	10.34	17.09	11.63	6.53	11.93	2.08	0.23	5.00	0.36	13.92
BC1mF2R	26–37	25.15	19.80	47.46	2.18	0	0	0	0	0	0	0	5.42
BC1pF2R	26–40	22.53	5.72	39.54	16.24	14.09	0.79	0.50	0	0	0	0	0.60
BC1mF3R	26–37	24.60	10.61	63.70	1.10	0	0	0	0	0	0	0	0
BC1pF3R	26–38	22.33	11.43	64.46	1.15	0.48	0	0	0	0	0	0	0.29
BC1mF4R	28–41	21.66	12.90	52.70	3.23	7.56	0	0	0.32	0	0	0	1.62
BC1pF4R	26–44	23.00	19.38	47.33	6.38	0.33	0	0	0	0	0	0.40	3.18

*RCN means the range of chromosome number*.

*At least 30 cells from each plant of the first- to fourth-generation progenies were analyzed*.

*BC1mF1R to BC1mF4R and BC1pF1R to BC1pF4R are the glyphosate-tolerant first- to fourth-generation progenies of the first backcross generation (BC1) obtained from wild Brassica juncea × F1R or F1R × wild B. juncea, respectively. F1R indicates the glyphosate-tolerant F1 hybrids obtained by wild B. juncea × glyphosate-tolerant transgenic oilseed rape. Progenitors in front of the × are always maternal plants, and progenitors after the × are always paternal plants*.

For the four-generation progenies from *B. napus* whose transgene is located on C-chromosome, the average chromosome numbers are shown in Table 2. The progeny had 28–44 chromosomes per individual plant in the first-generation (Supplementary Tables 4-9,4-10) and 26–42 in the second-generation (Supplementary Tables 4-11,4-12). In the third- and fourth-generation progeny, there were 26–41 (Supplementary Tables 4-13,4-14) and 28–38 chromosomes (Supplementary Tables 4-15,4-16), respectively. From the second- to fourth- generation progeny, the plants displayed a very particular chromosome number distribution with a large proportion (> 50% of cells in most cases) having 2*n* = 37 chromosomes.

**Table 2:** Average percentage of ovary cells with different chromosomes in the glufosinate-tolerant first- to fourth-generation progenies of the first backcross generation.

**Plant**	**RCN**	**Chromosome number**										
		**<36**	**36**	**37**	**38**	**39**	**40**	**41**	**42**	**43**	**44**	**>45**
BC1mF1L	28–44	5.08	2.42	11.50	12.70	9.17	24.62	5.94	10.12	6.78	6.23	6.23
BC1pF1L	29–43	12.84	4.72	14.75	12.22	12.83	13.36	10.82	1.64	9.36	0	7.48
BC1mF2L	26–42	25.14	6.64	43.47	10.73	4.35	3.62	1.09	4.38	0	0	0.60
BC1pF2L	27–42	25.05	15.34	32.65	7.08	1.96	5.21	9.93	1.14	0	0	1.65
BC1mF3L	29–38	18.52	10.81	61.70	9.45	0	0	0	0	0	0	0.48
BC1pF3L	26–41	19.79	12.33	55.71	3.77	2.70	2.83	0.26	0	0	0	2.60
BC1mF4L	28–38	20.92	17.76	53.07	6.77	0	0	0	0	0	0	1.50
BC1pF4L	29–38	19.91	15.35	61.44	3.04	0	0	0	0	0	0	0.26

*RCN means the range of chromosome number*.

*At least 30 cells from each plant of the first- to fourth-generation progeny were analyzed*.

*BC1mF1L to BC1mF4L and BC1pF1L to BC1pF4L are the glufosinate-tolerant first- to fourth-generation progenies of the first backcross generation (BC1) obtained from wild Brassica juncea × F1L or F1L × wild B. juncea, respectively. F1L indicates glufosinate-tolerant F1 hybrids obtained from wild B. juncea × glufosinate-tolerant transgenic oilseed rape. Progenitors in front of the × are always maternal plants, and progenitors after the × are always paternal plants*.

#### Chromosome Constitution

**Figure 6:**
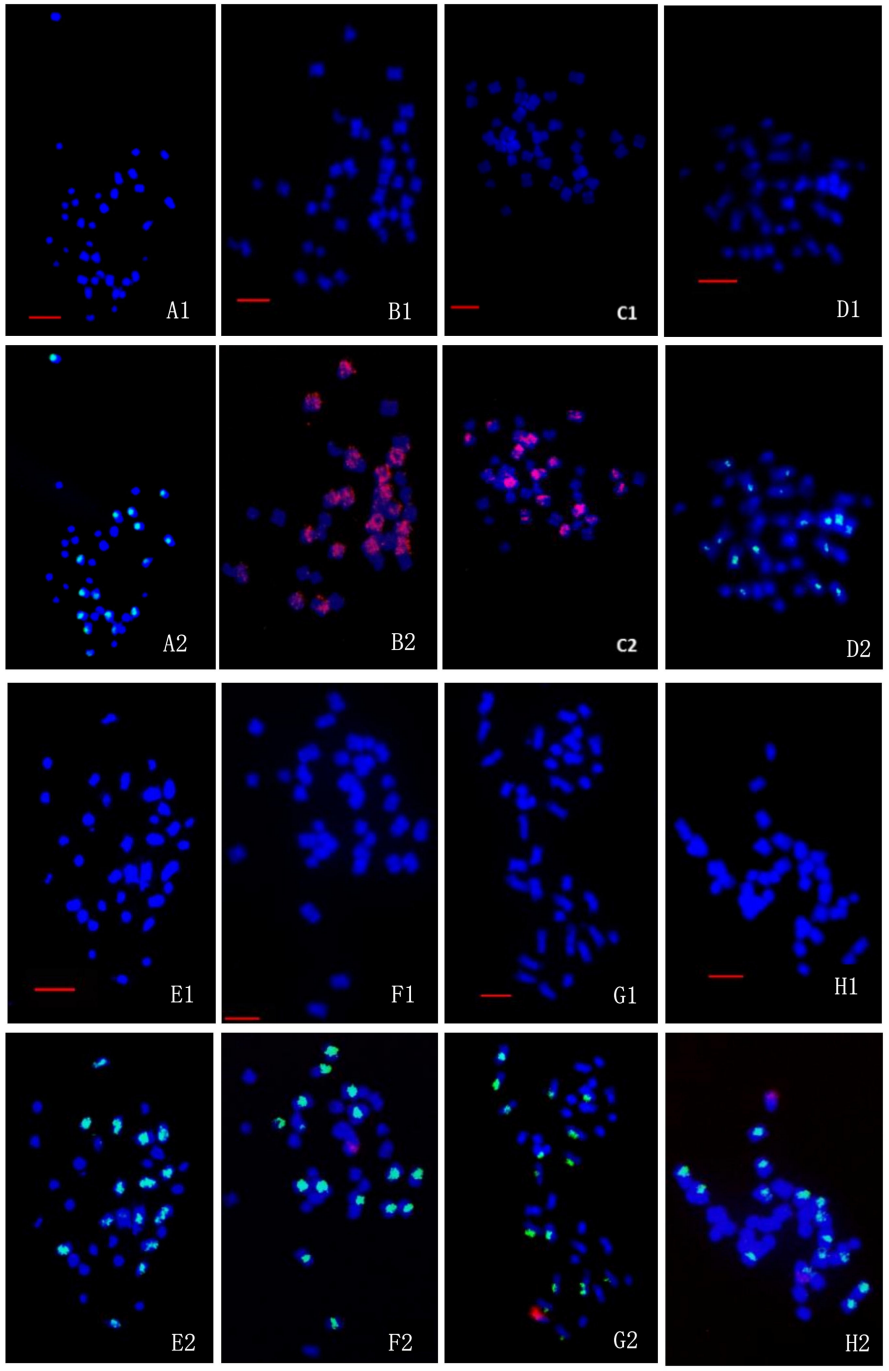
FISH/BAC-FISH analyses of the chromosome constitutions of the somatic cells of wild *Brassica juncea*, transgenic oilseed rape, and the fourth-generation progenies of BC1. **(A1–H1)** are the DAPI (blue) images for all chromosome, and **(A2–H2)** are merged images of the DAPI (blue), B chromosome probe (green), and C chromosome probe (red). **(A1-A2):** wild *B. juncea*: 20A + 16B. **(B1-B2):** transgenic oilseed rape with glyphosate-tolerant gene: 20A+18C. **(C1-C2):** transgenic oilseed rape with glufosinate-tolerant gene: 20A + 18C. **(D1-D2):** the cells of BC1mF4R with 36 chromosomes: 20A + 16B. **(E1-E2):** the cells of BC1pF4R with 36 chromosomes: 20A + 16B. **(F1-F2):** the cells of BC1mF4L with 37 chromosomes: 20A + 16B + 1C. **(G1-G2):** the cells of BC1pF4L with 37 chromosomes: 20A + 16B + 1C. **(H1-H2):** the cells of BC1pF4L with 37 chromosomes: 20A + 15B + 2C. Green fluorescent signals from probe of *B. nigra*, red fluorescent signals from C genome-specific BAC BoB014O06. BC1mF4R and BC1pF4R indicate the glyphosate-tolerant fourth-generation progenies of the first backcross generation (BC1) obtained from wild *Brassica juncea* × F1R or F1R × wild *B. juncea*, respectively. B C1mF4L and BC1pF4L indicate the glufosinate-tolerant fourth-generation progenies of the first backcross generation (BC1) obtained from wild *Brassica juncea* × F1L or F1L × wild *B. juncea*, respectively. F1R and F1L indicate glyphosate- or glufosinate-tolerant F1 hybrids obtained from wild *B. juncea* × glyphosate- or glufosinate-tolerant transgenic oilseed rape. Progenitors in front of the × are always the maternal plants, and the progenitors after the × are always the paternal plants. All bars: 5 μm.

A and B chromosomes of wild *B. juncea* were easily distinguished by using the labeled *B. nigra* genomic DNA probe (green for B) and A and C chromosomes of transgenic oilseed rape by using the labeled *B. oleracea* BoB014O06 probe (red for C). The chromosomes from the A, B, and C genomes in the progeny of BC1 were identified by dual-color FISH, with the labeled *B. nigra* DNA (green for B) and BoB014O06 probes (red for C). According to the FISH observations, the genomic constitutions of the wild *B. juncea* and the transgenic oilseed rape were 20A + 16B (Figures 6A1,A2) and 20A + 18C (Figures 6B1,B2, glyphosate-tolerant transgene located on A-chromosome; Figures 6C1,C2 glufosinate-tolerant transgene located on C-chromosome), respectively. The genomic constitutions of the 36-chromosome cells of either BC1mF4R (Figures 6D1,D2) or BC1pF4R (Figures 6E1,E2) were 20A + 16B. However, the genomic constitutions of the cells with 37 chromosomes of either BC1mF4L (Figures 6F1,F2) or BC1pF4L (Figures 6G1,G2) were 20A + 16B + 1C. Occasionally, they were 20A + 15B + 2C (Figures 6H1,H2), as was found in two of 109 cells observed of BC1pF5L plants. The fourth-generation progenies from *B. napus* whose transgene located on C-chromosome always carried one, or occasionally two additional oilseed rape C-chromosomes labeled by C-genome specific BAC BoB014O06.

#### Chromosome Behavior During Meiosis

**Figure 7:**
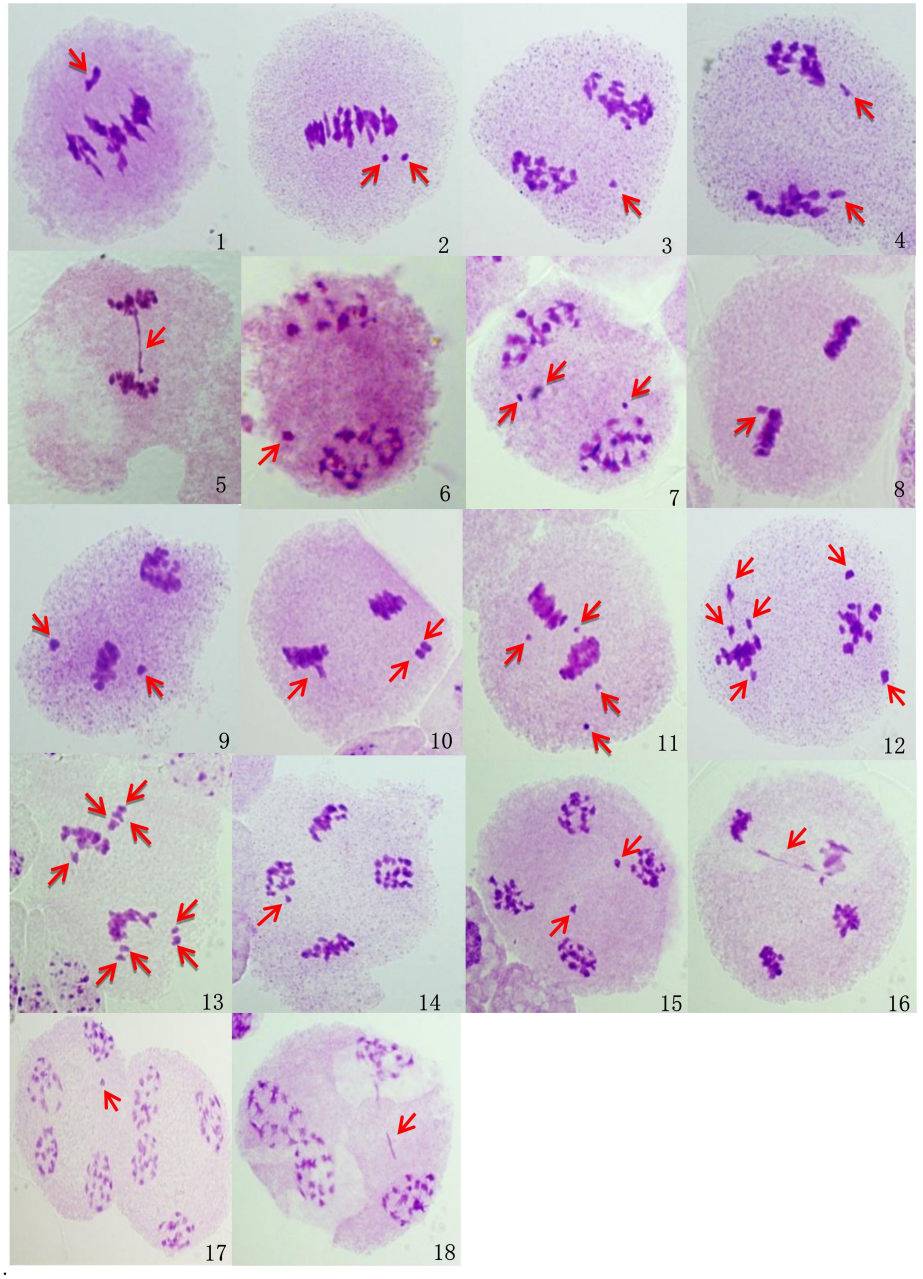
Microscopic observations of the abnormal meiosis of pollen mother cells in BC1mF4R. Images were taken using a light microscope (ZEISS, imager. M2, 100 ×). (1-2). **Metaphase I:** 1. One lagging chromosome; (2). two lagging chromosomes. (3-5). **Anaphase I:** 3. one lagging chromosome; 4. two lagging chromosomes; 5. one chromatid bridge. (6-7). **Telophase I:** 6. one micronucleus; (7). three micronuclei. (8-13). **Metaphase II:** (8). one lagging chromosome; (9). two lagging chromosomes; (10). three lagging chromosomes; (11). four lagging chromosomes; (12). six lagging chromosomes; (13). eight lagging chromosomes. (14-16). **Anaphase II:** (14). one lagging chromosome; (15). two lagging chromosomes; (16). one chromatid bridge. (17-18). **Telophase II:** (17). one micronucleus; (18). one chromatid bridge. BC1mF4R indicates the glyphosate-tolerant fourth-generation progenies of the first backcross generation (BC1) obtained from wild *Brassica juncea* × F1R. F1R indicates the glyphosate-tolerant F1 hybrids obtained from wild *B. juncea* × glyphosate-tolerant transgenic oilseed rape. Progenitors in front of the × are always the maternal plants, and progenitors after the × are always the paternal plants. Red arrows indicate the abnormal meiosis of pollen mother cells.

Abnormal meiosis was observed in the HT fourth-generation progenies of BC1 from *B. napus* whose transgene is located on either A- or C-chromosome. Lagging chromosomes were observed in metaphase I and II. These lagging chromosomes did not pair normally and were not arranged on the metaphase plate. Lagging chromosomes and chromatid bridges, which were mainly induced by chromosome inversions in anaphase I and II, were frequently observed. Some lagging chromosomes failed to enter the polar region where the other chromosomes started to condense; these lagging chromosomes in anaphase I and II formed micronuclei in telophase I and II and were recorded at a low frequency. Occasionally, the chromatid bridge in anaphase II lasted to telophase II, and a chromatid bridge was observed in telophase II. Although the glyphosate- or glufosinate-tolerant fourth-generation progeny had consistent abnormal behaviors, the chromosome pairings in the glufosinate-tolerant fourth-generation progeny from the cultivar having the transgene located on the C-chromosome were much more highly irregular than the glyphosate-tolerant fourth-generation progeny from the cultivar having the transgene located on the A-chromosome (Table 3, Figure 7, Supplementary Figures 3-1–3-3).

**Table 3:** Frequency of meiotic abnormalities in the glyphosate- or glufosinate-tolerant fourth-generation progenies of the first backcross generation.

**Stage**	**BC1mF4R**		**BC1pF4R**		**BC1mF4L**		**BC1pF4L**	
	**NCO**	**F (%)**	**NCO**	**F (%)**	**NCO**	**F (%)**	**NCO**	**F (%)**
MetaphaseI	842	28.76	692	44.57	1,291	62.16	1,465	44.63
AnaphaseI	573	11.90	784	27.07	661	60.36	736	64.52
TelophaseI	795	1.59	887	2.35	807	11.60	647	10.51
MetaphaseII	942	36.80	1,068	47.73	763	50.95	768	46.11
AnaphaseII	759	9.24	836	15.90	925	72.63	692	42.35
TelophaseII	1,054	3.77	1,100	2.93	935	21.13	980	14.61

*NOC means the number of cells observed. F means the frequency of meiotic abnormalities*.

*BC1mF4R and BC1pF4R indicate the glyphosate-tolerant fourth-generation progenies of the first backcross generation (BC1) obtained from wild Brassica juncea × F1R or F1R × wild B. juncea, respectively. BC1mF4L and BC1pF4L indicate the glufosinate-tolerant fourth-generation progenies of the first backcross generation (BC1) obtained from wild Brassica juncea × F1L or F1L × wild B. juncea, respectively*.

*F1R and F1L indicate glyphosate- or glufosinate-tolerant F1 hybrids obtained from wild B. juncea × glyphosate- or glufosinate-tolerant transgenic oilseed rape. Progenitors in front of the × are always maternal plants, and progenitors after the × are always paternal plants*.

The meiotic indices of the BC1mF4R and BC1pF4R were 96.20 and 97.10%, respectively, which indicates stable inheritance. In contrast, the values for BC1mF4L and BC1pF4L were 78.90 and 85.34%, respectively, which indicated that they were genetically unstable.

### The Persistence of the C-Chromosome Regions of *B. napus* in the Progenies of BC1

**Figure 8:**
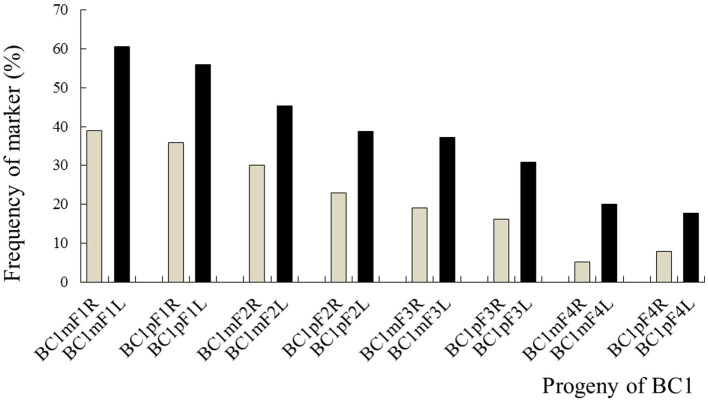
Frequency of the marker specific to the C-chromosome of *Brassica napus* in the first- to fourth-generation progenies of BC1. BC1mF1R to BC1mF4R and BC1pF1R to BC1pF4R are the glyphosate-tolerant first- to fourth-generation progenies of the first backcross generation (BC1) obtained from wild *Brassica juncea* × F1R or F1R × wild *B. juncea*, respectively. BC1mF1L to BC1mF4L and BC1pF1L to BC1pF4L are the glufosinate-tolerant first- to fourth-generation progenies of the first backcross generation (BC1) obtained from wild *Brassica juncea* × F1L or F1L × wild *B. juncea*, respectively. F1R or F1L are the glyphosate- or glufosinate-tolerant F1 hybrids obtained from wild *B. juncea* × glyphosate- or glufosinate-tolerant transgenic oilseed rape. Progenitors in front of the × are always the maternal plants, and progenitors after the × are always the paternal plants.

**Figure 9:**
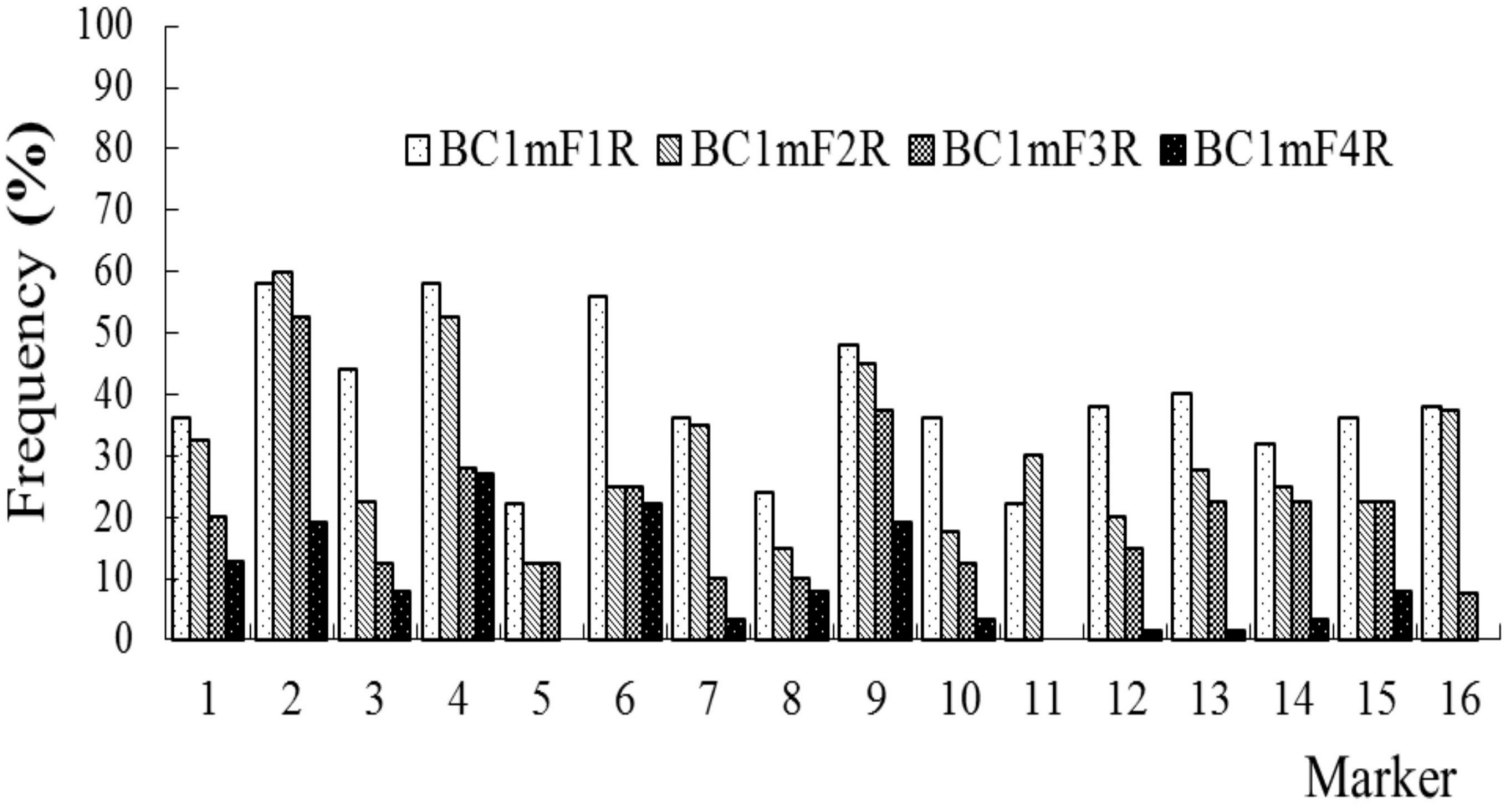
Frequency of the marker specific to the C-chromosome of *Brassica napus* in the first (BC1mF1R)- to fourth (BC1mF4R)-generation progenies of BC1. BC1mF1 R to BC1mF4R are the glyphosate-tolerant first- to fourth-generation progenies of the first backcross generation (BC1) obtained from wild *Brassica juncea* × F1R. F1R indicates the glyphosate-tolerant F1 hybrids obtained from wild *B. juncea* × glyphosate-tolerant transgenic oilseed rape. Progenitors in front of the × are always the maternal plants, and progenitors after the × are always the paternal plants.

**Figure 10:**
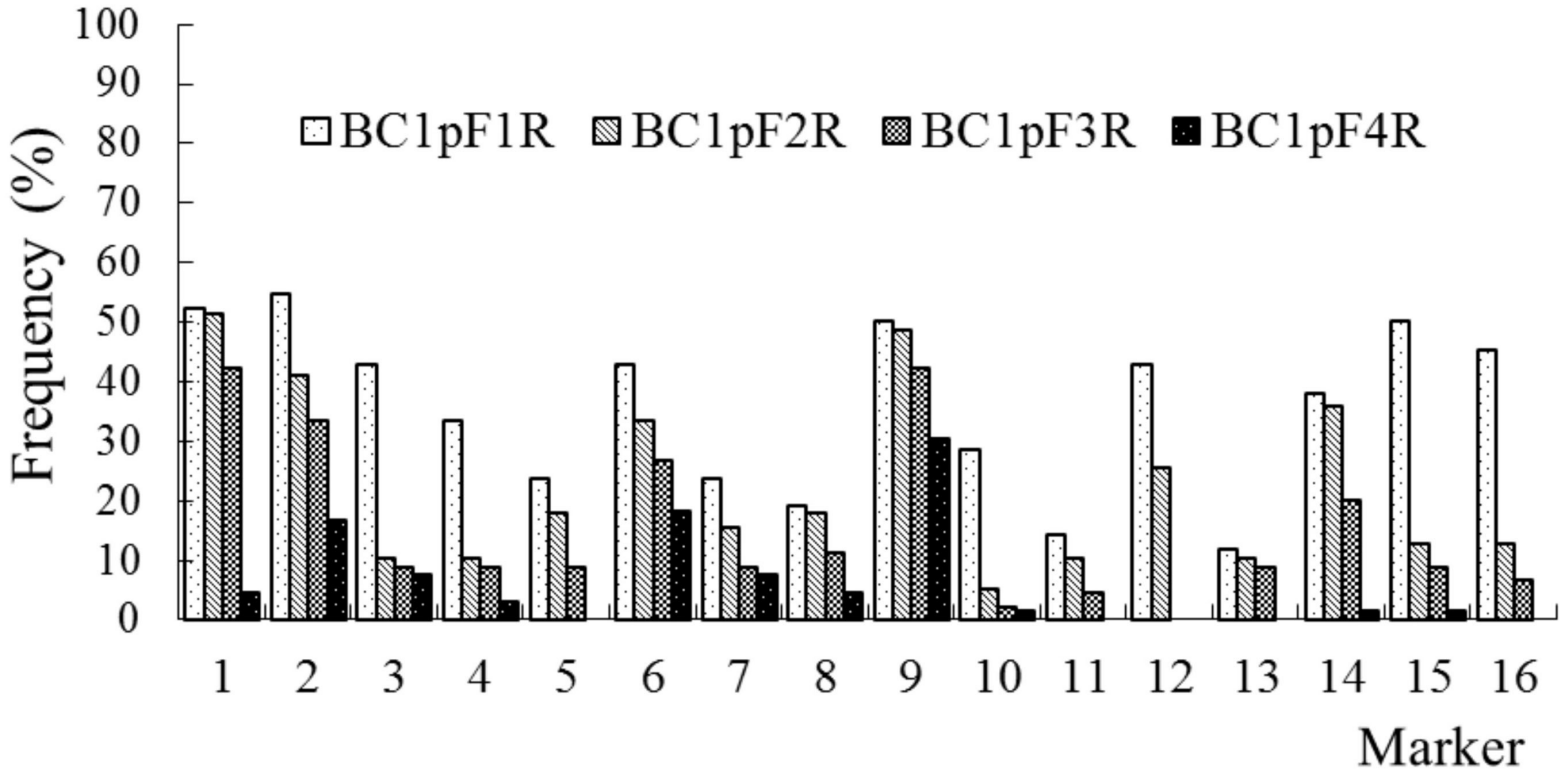
Frequency of the marker specific to the C-chromosome of *Brassica napus* in the first (BC1pF1R)- to fourth (BC1pF4R)-generation progenies of BC1. BC1pF1R to BC1pF4R are the glyphosate-tolerant first- to fourth-generation progenies from the first backcross generation (BC1) of F1R × wild *Brassica juncea*. F1R indicates the glyphosate-tolerant F1 hybrids obtained from wild *B. juncea* × glyphosate-tolerant transgenic oilseed rape. Progenitors in front of the × are always the maternal plants, and progenitors after the × are always the paternal plants.

**Figure 11:**
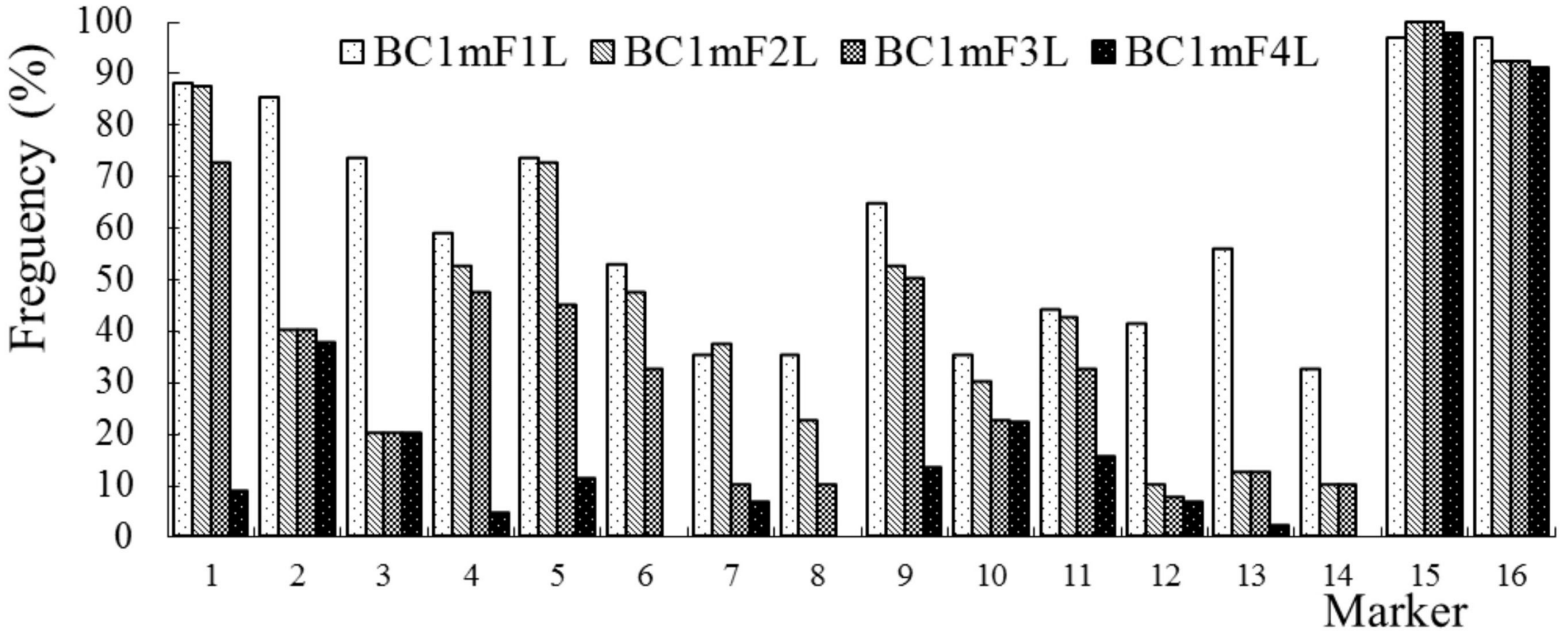
Frequency of the marker specific to the C-chromosome of *Brassica napus* in the first (BC1mF1L)- to fourth (BC1mF4L)-generation progenies of BC1. BC1mF1L to BC1mF4L are the glufosinate-tolerant first- to fourth-generation progenies of the first backcross generation (BC1) obtained from wild *Brassica juncea* × F1L. F1L indicates the glufosinate-tolerant F1 hybrids obtained from wild *B. juncea* × glufosinate-tolerant transgenic oilseed rape. Progenitors in front of the × are always the maternal plants, and progenitors after the × are always the paternal plants.

**Figure 12:**
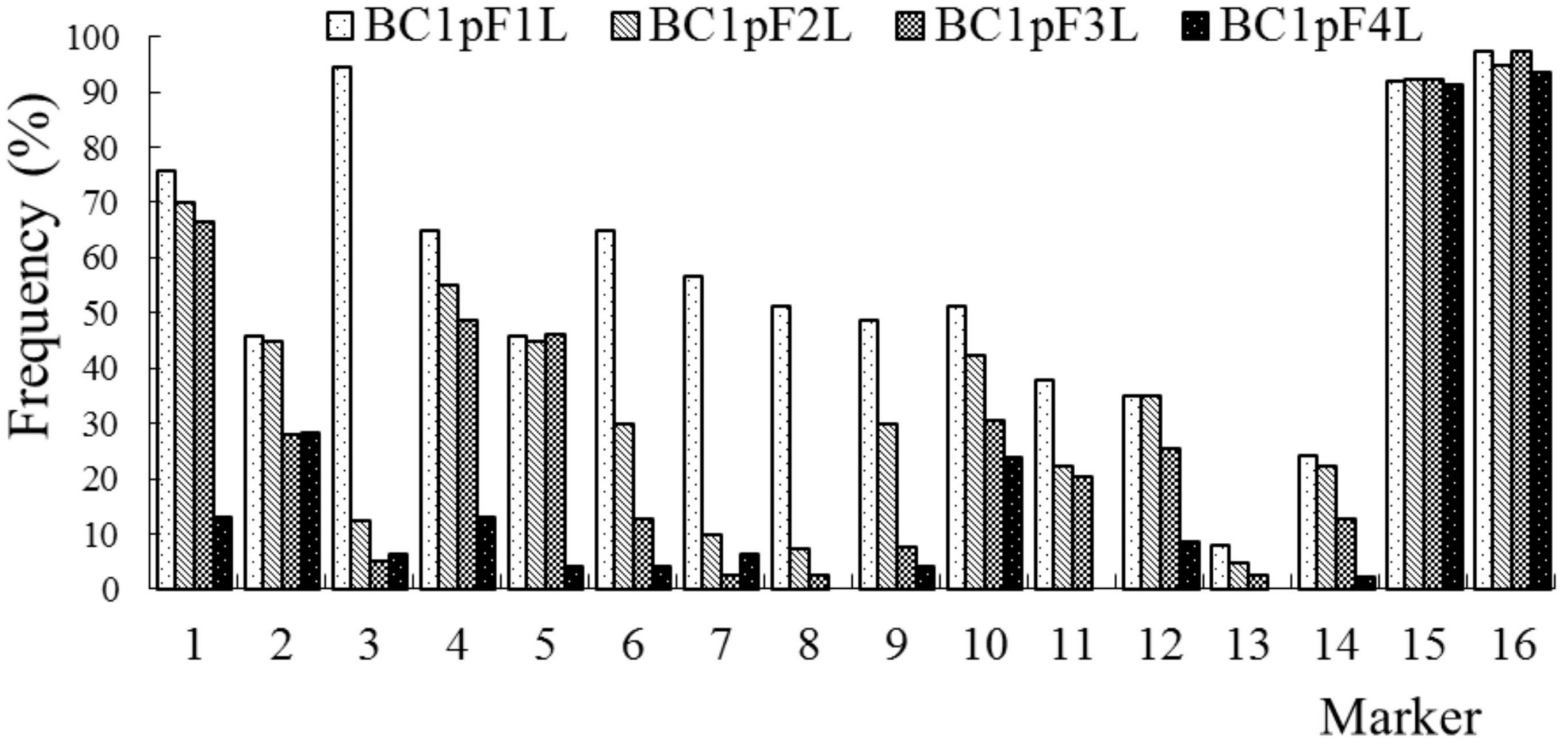
Frequency of the marker specific to the C-chromosome of *Brassica napus* in the first (BC1pF1L)- to fourth (BC1pF4L)-generation progenies of BC1. BC1pF1L to BC1pF4L are the glufosinate-tolerant first- to fourth-generation progenies of the first backcross generation (BC1) obtained from F1L × wild *Brassica juncea*. F1L indicates the glufosinate-tolerant F1 hybrids obtained from wild *B. juncea* × glufosinate-tolerant transgenic oilseed rape. Progenitors in front of the × are always the maternal plants, and progenitors after the × are always the paternal plants.

The average frequencies of the markers specific to the C-chromosome in the different progenies of the BC1 are shown in Figure 8. The frequencies of the markers detected in the glyphosate-tolerant progeny from the cultivar having the transgene located on the A-chromosome were consistently lower than in glufosinate-tolerant progeny from the cultivar having transgene located on the C-chromosome, in all generations. For the glyphosate-tolerant progeny, the marker detection frequency decreased with the increasing self-pollination of BC1. In BC1mF4R and BC1pF4R, 13 and 11 of the 16 markers were still detected at various frequencies, respectively (Figures 9, 10). For the glufosinate-tolerant progeny, the marker detection frequency decreased from the first to fourth generations, except for markers 15 and 16, whose frequencies were above 91 % (Figures 11, 12); these two markers were from chromosome C8 of *B. napus* that carried the glufosinate tolerance gene (Supplementary Material 1, Supplementary Table 2). However, the marker 15 was detected at <8% frequency, and the marker 16 was not observed in the glyphosate-tolerant fourth progeny.

## Discussion

Successful gene flow from transgenic species to their wild relatives depends on their introgression ability, including the inheritance stability of the transgenes and fitness among hybrid progenies; these may be determined by genetic factors related to chromosome structures and transgene insertion locations.

### Emergence

Although the emergence percentage of the majority of the first- and second-generation progeny was lower than that of the wild *B. juncea* population, with a trend toward an increase as generations advanced, sufficient seedlings were recruited (above 85%) to secure their establishment and the production of enough seed for succeeding generations. Therefore, the emergence percentage did not limit the production of successive progeny.

### Herbicide Tolerance Segregation in Progeny

It was speculated that gene introgression from *B. napus* to its relatives *B. rapa* or *B. juncea* occurs more easily when the transgene is originally carried by the A genome of the oilseed rape than by the C genome (Metz et al., 1997; Lu et al., 2002; Liu et al., 2010; Guan et al., 2020). The high degree of homology between the A-genomes of *B. napus* and wild *B. juncea* suggests that an exchange of genes located on the A-genome could occur relatively unimpeded by recombination (Frello et al., 1995; Liu et al., 2010). However, as the C-chromosomes have no homologous partners during meiosis, a transgene located on the C-genome of *B. napus* should be transmitted at a low frequency to the gametes, and should be lost during meiosis with an increased number of backcrosses, due to the irregular transmission of C-chromosomes to gametes (Metz et al., 1997; Lu et al., 2002). In this investigation, the glyphosate tolerance gene was located on an A-chromosome; thus, this trait was easily transferred from the *B. napus* to the wild *B. juncea* and stably inherited in subsequent generations of BC1, regardless of whether the wild *B. juncea* parent was female or male under glyphosate selection. However, the progenies, from *B. napus* whose glufosinate tolerance gene was located on C-chromosome, differed with respect to segregation into HT and herbicide-sensitive plants. HT trait was transferred at lower percentages in progeny of BC1 from *B. napus* whose transgene located on C- than on A-chromosome. Therefore, if the transgenic crop is released for cultivation, it should have lower risk of its C-genome linked transgene to flow and introgress in wild *B. juncea* compared with an A-genome-linked transgene under herbicide selection.

It should be noted that we did not address the persistence of the herbicide resistance trait in the absence of herbicide pressure when the transgene was located on a C-chromosome. This situation would be relevant in the field if the tolerant variety were no longer grown. In the future, it may be possible to minimize potential ecological risk of transgenes persisting in wild populations in the environment by using specific insertion sites in the genomes, through novel molecular techniques.

### Fitness-Associated Traits of Backcross Progeny

The likelihood of transgenes spreading by introgression from crops into the weedy populations of related species depends in part on the fitness of the first and successive generations of hybrids (Hauser et al., 1998a,b; Gueritaine et al., 2002; Jenczewski et al., 2003; Liu et al., 2013a,b, 2018). Although the reproductive fitness of the first backcross generation of *B. napus* and the wild relative *B. rapa* was significantly lower compared with the parental generation, the first backcross generation hybrids had an enhanced reproductive fitness compared with the first-generation hybrids (Ammitzbøll et al., 2005). Furthermore, the pollen fertility and seed production of the third backcross generation plants were as great as those of *B. rapa* (Snow et al., 1999). For *B. napus*–*R. raphanistrum* hybrids, the female fertility increased continuously over successive backcross generations (Chèvre et al., 1997).

Gene flow of transgenic *B. napus* carrying herbicide tolerance transgenes located on either A- or C- genomes might occur in the field *via* the crossing of F1 hybrids with wild *B. juncea* and the self-pollination of the first backcross, due to continued increased fitness over successive generations. However, the risks associated with gene flow from transgenic *B. napus* carrying herbicide tolerance transgenes located on A-chromosomes could be much greater than those on C-chromosomes, because their third- and fourth-generation progenies in comparison performed significantly better, especially with respect to seeds per silique. The fitness differences between these two kinds of progenies may be attributed to the different genomic constitutions. It may be due to the additional C chromosome of *B. napus* which herbicide tolerance gene located on was incorporated into the genome of *B. juncea*. The results implied that the fecundity of progeny, which is the most important fitness-associated trait, was highly dependent on chromosome constitution.

Previous research demonstrated that the cytoplasm had a significant effect on all measured characteristics of the backcross hybrids between the transgenic oilseed rape and wild radish (Gueritaine et al., 2002). The fitness of the progeny was similar for most of the components, regardless of whether the wild *B. juncea* was the maternal or paternal progenitor. This may be attributed to the progeny of wild *B. juncea* × F1 (wild *B. juncea* × *B. napus*) and F1 (wild *B. juncea* × *B. napus*) × wild *B. juncea*, having the same wild *B. juncea* cytoplasm. Therefore, these are two possible routes for transgene movement in wild *B. juncea*, as either maternal or paternal plants. However, if F1 hybrid pollen is not abundant enough, subsequent hybrids are likely to occur, as pollen will be obtained from the wild *B. juncea*, which could result in the movement of transgenes toward wild *B. juncea* as the male. This route is more easily accomplished than the reciprocal.

### Chromosome Number and Constitution in the BC1 Progeny

Mixoploidy is a common phenomenon in distant hybridization (Li et al., 1995, 1998; Li and Heneen, 1999; Hua et al., 2006). We confirmed that the first- to fourth-generation progenies of BC1 from *B. napus* whose transgene was located on either A- or C-chromosome were mixoploids. The F1 hybrids between *B. napus* and wild *B. juncea* had 37 chromosomes (Tsuda et al., 2012). The first backcross generation (BC1) between F1 hybrids and wild *B. juncea* should have a varying chromosome number of 20 + 8 (AA+ B) +0–8 (B) + 0–9 (C) due to genetic imbalance. The variable chromosome numbers in the somatic tissues of the progeny plants of BC1 were attributed to the possible separation of the parental genomes during mitotic and meiotic divisions of some hybrid cells (Li et al., 1995, 1998; Li and Heneen, 1999; Hua et al., 2006). The presumed abnormal mitotic separation and elimination of the C-genome of *B. napus* provided an explanation for the somatic components in the first- to fourth-generation progenies of BC1. The abnormal meiosis of the PMC, confirmed during meiosis of the HT fourth-generation progenies from *B. napus* having the transgene on A- and C-chromosomes, also contributed to this result. The present research verified the complicated evolution process of hybrids of *B. napus* and wild *B. juncea*. Large differences in the chromosome constitutions of the hybrids between the transgenic crops and their weedy relatives could complicate environmental risk assessments for transgenic crops.

Although mixoploidy results in the cytological instability of the BC1 progeny from the transgenic oilseed rape and wild *B. juncea*, the progeny displayed increased fecundity and fitness progressively, with the narrowing of the chromosome variation across the self-pollinating generations of the BC1. Therefore, the mixoploid progeny from cultivars with the transgene located on either A- or C-genomes could survive under herbicide selection pressure. Regardless of the significant chromosome disorders, and despite the apparent lack of stabilized introgression, the transgenes located on the A-genome and on the C-genome might be maintained for a very long time in populations subjected to herbicide selection pressure.

The large proportion of cells of the progeny from *B. napus* whose transgene located on A-chromosome had AABB genomic constitution, which was the same as *B. juncea*. However, the large proportion of cell of the progeny from *B. napus* whose transgene located on C-chromosome had AABB+1C genomic constitution. These results were confirmed by BAC-FISH and SSR marker analyses. One alien C-chromosome, carrying the glufosinate tolerance gene, was added into the fourth-generation progeny in the presence of glufosinate selection although this trait was inherited in a non-Mendelian fashion. Thus, chromosome additions are also an avenue for introgression of the C-genome into wild *B. juncea*. In previous research, the glufosinate-resistant progeny of *B. napus* and *R. raphanistrum* was presumed to carry a supernumerary chromosome from oilseed rape that conveyed herbicide resistance, when subjected to herbicide selection pressure (Al Mouemar and Darmency, 2004). Introgression from *B. napus* to *B. juncea* could also be accomplished by C-chromosome additions (Frello et al., 1995). The current research proved directly that the C-chromosome, carrying a herbicide tolerance gene, was retained in the progeny of the transgenic oilseed rape and wild relatives, as a supernumerary chromosome, although we did not assess if the C-chromosome was transmitted as a complete or incomplete structure. The C-chromosome in HT progeny from transgenic *B. napus* was labeled by *B. oleracea* BoB014O06 probe clearly for the first time. The clone *B. oleracea* BoB014O06 hybridizes to the C-chromosomes and allows visualization of the C-genome *B. napus*. It is highly efficient in distinguishing A and C *Brassica* chromosomes (Leflon et al., 2006; Cui et al., 2012).

Besides the location of the transgene, the genotype of the transgenic oilseed rape may influence the genome evolution of hybrids. In the present study, the two transgenic rapeseed lines were generated in different genotypes. Since polymorphism of the wild *B. juncea* was not a factor here, the genotypic differences should be due to the transgenic oilseed rape. Chèvre et al. (2007) found that the numerical distributions of the chromosomes from the first backcross generations of different genetically modified oilseed rape lines and *R. raphanistrum* were clearly different, according to the flow cytometry estimations. Homologous pairing during meiosis in the interspecific hybrids between *B. napus* and *B. carinata* was affected by the hybrid genotype (Mason et al., 2011). Besides genotypes of transgenic rapeseed lines, multiple genotypes and high genetic diversity of wild *B. juncea* in natural environments may complicate the genome evolution of hybrids (Dong et al., 2018; Sun et al., 2018). Further research should be conducted on the effects that genotypes both from transgene oilseeds rape and from wild *B. juncea* have on the evolution of hybrids.

### Persistence of C-Chromosome Regions of *B. napus* in Progeny

Partial chromosomal homology among the A, B, and C genomes, and higher homology between A and C genome than A and B genome, has been documented (Hosaka et al., 1990; Kerlan et al., 1993; Frello et al., 1995; Hasterok et al., 2005; Town et al., 2006; Yang et al., 2006; Ge and Li, 2007; Akaba et al., 2009; Mason et al., 2011; Navabi et al., 2011). Therefore, allosyndesis within A–C or A-B genome chromosomes of the backcross generation between *B. napus* and wild *B. juncea* may occur and cause the persistence of C-chromosome regions in these backcross generations (Tsuda et al., 2012; Guan et al., 2020). However, when the selection stress was absent, C-genome-specific markers were rarely found in the BC2 and BC3 generations from the conventional *B. napus* and wild *B. juncea* (Tsuda et al., 2012) and BC2–BC5 generations from the *B. napus* with *GFP* and *Bt Cry1Ac* transgene on A-chromosome and *B. juncea* (Guan et al., 2020).

In the presence of herbicide selection, for the progenies from *B. napus* carrying the transgene on the A genome, all 16 markers located on C2 to C8 were transferred to the first- and second-generation progeny of BC1, and 15 markers transferred to the third-generation progeny. Three markers for BC1mF4R and five markers for BC1pF4R were not detected in the fourth-generation progeny, while the rest of the markers remained and were detected at <31% frequency. Combined with the 20 A + 16 B of the genomic constitutions of the 36 chromosomes cells of these fourth progenies of BC1, the persisting C-chromosome from *B. napus* with the transgene located on the A genome of *B. napus*, were integrated into A genome chromosomes of wild *B. juncea* by homologous recombination at high possibility, or B genome chromosomes at low possibility.

For the progenies from *B. napus* whose transgene was located on C8 chromosome, all 14 markers located on C2 to C7 were transferred to the first-, second-, and third-generation progeny of BC1. Three markers for BC1mF4L and two markers for BC1pF4L were not detected in the fourth-generation progeny, while the rest of the markers remained and were detected at <38% frequency. Therefore, the persistent C-chromosome regions located on C2-C7 chromosomes of *B. napus* on which transgene did not locate may be due to the integration of C-chromosome regions into the A genome chromosomes of wild *B. juncea* at high possibility, or B genome chromosomes at low possibility.

However, markers of 15 and 16, located on the same chromosome with transgene, were transmitted to the first to fourth progenies of the BC1, at more than 91% frequency. Combined with the 20 A + 16 B + 1 C of the genomic constitutions of the 37 chromosome cells of these progenies, C-chromosome with a herbicide tolerance transgene was added into the progeny under herbicide selection as a supernumerary chromosome. C-chromosome additions could occur during genetic transmission under herbicide selection besides A-C, or A-B chromosomal recombination. Herbicide selection may be a powerful force to drive the introgression of C chromosomes from *B. napus* into wild *B. juncea*.

The original insertion site of the transgene affects introgression under herbicide selection. The assessment of the transgene frequency and survival fitness of the progenies indicates a lower gene flow risk for the transgenes located on the C- rather than the A-chromosomes of *B. napus*. The results confirm the complex nature of the genetic processes occurring during self-pollination of the backcross generations of the transgenic oilseed rape and wild *B. juncea*. The findings suggest that the ecological risks for gene flow from the transgenic crops to their wild relatives can be mitigated through the breeding of transgenic allopolyploid crops, in which the transgene is inserted into an alien chromosome.

## Key Message

The C-chromosome from *Brassica napus* carrying a herbicide TOLERANCE transgene introgressed into wild *B. juncea* as a supernumerary, alien chromosome under herbicide selection, inducing genetic instability in progeny thus reducing the probability of gene flow from the transformed crop to the wild congener.

## Data Availability Statement

The DNA sequence of A1 chromosome (cp4-epsps located) was retrieved from the NCBI Database, NCBI Reference Sequence: NC_027757.1, Gene bank accessions: NC_027757. The DNA sequence of C8 chromosome (pat located) was retrieved from the NCBI Database, NCBI Reference Sequence: NC_027774.1, Gene bank accessions: NC_027774. The left flanking DNA sequence of promoters FMV35S was retrieved from the NCBI Genome Database, NCBI Reference Sequence: GX257885.1, Gene bank accessions: GX257885. The left flanking DNA sequence of promoters CaMV35S was retrieved from the NCBI Genome Database, NCBI Reference Sequence: FJ154954.1, Gene bank accessions: FJ154954. The oilseed rape whole genome sequence was retrieved from the NCBI Genome Database, Gene bank accessions: GCA_000686985.2. The other details of these DNA sequences are available in Supplementary Materials 1. Further inquiries can be directed to the corresponding author/s.

## Author Contributions

XS, HL, ZS, and YW performed the statistical analysis and drafted the manuscript. JY, YZ, AZ, QZ, JW, and QB participated in the data collection. XS and SQ designed the research. All authors have read and approved the final manuscript.

## Conflict of Interest

The authors declare that the research was conducted in the absence of any commercial or financial relationships that could be construed as a potential conflict of interest.
