# Supplementary material for: Gene Flow Risks From Transgenic Herbicide-Tolerant Crops to Their Wild Relatives Can Be Mitigated by Utilizing Alien Chromosomes
Source: Front Plant Sci. 2021 Jun 11;12:670209. doi: 10.3389/fpls.2021.670209 (PMC8231706; doi:10.3389/fpls.2021.670209)
Supplement: Supplementary file 1 [file Data_Sheet_1.zip › Supplementary Figure S1.pdf]

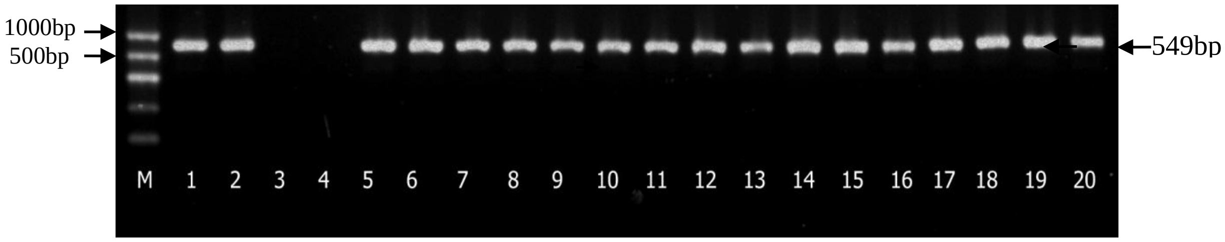

M: marker DL2000; **1-2**: Glyphosate tolerant transgenic oilseed rape; **3-4**: Wild *B. juncea*; **5-6**: BC1mF1R;

7-8: BC1pF1R, **9-10**:BC1mF2R; **11-12**: BC1pF2R; **13-14**: BC1mF3R; **15-16**: BC1pF3R; **17-18**: BC1mF4R;

19-20: BC1pF4R

FIGURE S 1-1 PCR amplification of *cp4-epsps* gene fragment from transgenic glyphosate-tolerant oilseed rape, and the first to fourth generation progenies of BC1

BC1mF1R to BC1mF4R and BC1pF1R to BC1pF4R are the glyphosate-tolerant first to fourth generation progenies of the first backcross generation (BC1) obtained from wild *Brassica juncea* × F1R or F1R × wild *B. juncea*, respectively. F1R indicates the glyphosate-tolerant F1 hybrids obtained from wild *B. juncea* × glyphosate-tolerant transgenic oilseed rape. Progenitors in front of the × are always maternal plants, and progenitors after the × are always paternal plants.

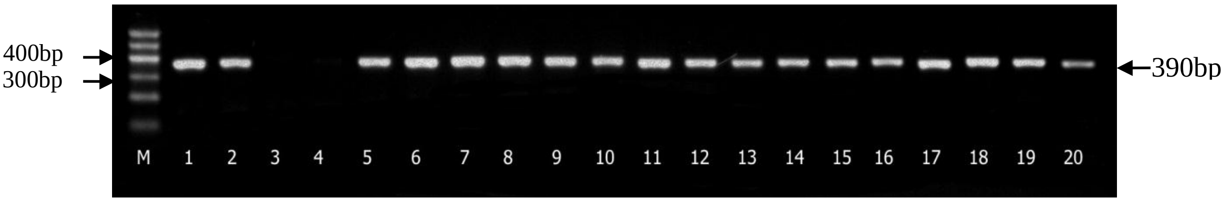

M: marker DL2000; **1-2**: Glufosinate tolerant transgenic oilseed rape; **3-4**: Wild *B. juncea*; **5-6**: BC1mF1L;

7-8: BC1pF1L, **9-10**:BC1mF2L; **11-12**: BC1pF2L; **13-14**: BC1mF3L; **15-16**: BC1pF3L; **17-18**: BC1mF4L;

19-20: BC1pF4L

FIGURE S1-2 PCR amplification of *pat* gene fragment from transgenic glufosinate oilseed rape, and the first to fourth generation progenies of BC1

BC1mF1L to BC1mF4L and BC1pF1L to BC1pF4L are the glufosinate-tolerant first to fourth generation progenies of the first backcross generation (BC1) obtained from wild *Brassica juncea* × F1L or F1L × wild *B. juncea*, respectively. F1L indicates the glufosinate-tolerant F1 hybrids obtained from wild *B. juncea* ×glufosinate-tolerant transgenic oilseed rape. Progenitors in front of the × are always maternal plants, and progenitors after the × are always paternal plants.
