# Supplementary material for: Gene Flow Risks From Transgenic Herbicide-Tolerant Crops to Their Wild Relatives Can Be Mitigated by Utilizing Alien Chromosomes
Source: Front Plant Sci. 2021 Jun 11;12:670209. doi: 10.3389/fpls.2021.670209 (PMC8231706; doi:10.3389/fpls.2021.670209)
Supplement: Supplementary file 1 [file Data_Sheet_1.zip › Supplementary Figure S2.pdf]

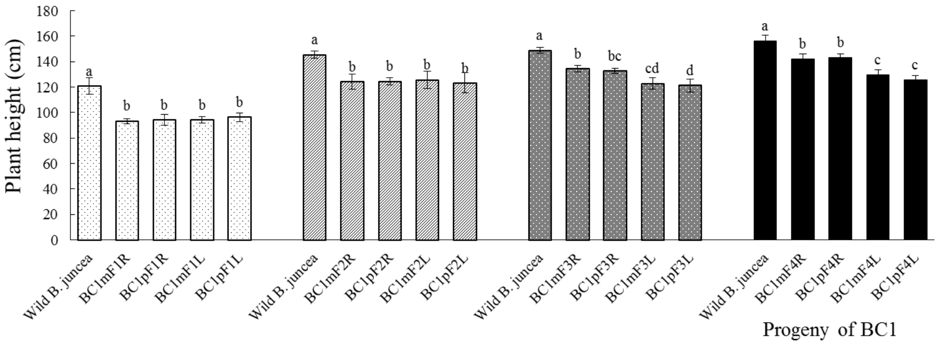

FIGURE S2-1 Plant height of the first to the fourth generation progenies of BC1

Data were shown as the mean ± SE. Different letters in the different progenies of the same generation indicate significant differences using Duncan's multiple range test, $P < 0.05$. BC1mF1R to BC1mF4R and BC1pF1R to BC1pF4R are the glyphosate-tolerant first to fourth generation progenies of the first backcross generation (BC1) obtained from wild *Brassica juncea* × F1R or F1R × wild *B. juncea*, respectively. BC1mF1L to BC1mF4L and BC1pF1L to BC1pF4L are the glufosinate-tolerant first to fourth generation progenies of the first backcross generation (BC1) obtained from wild *Brassica juncea* × F1L or F1L × wild *B. juncea*, respectively. F1R and F1L indicate glyphosate- or glufosinate-tolerant F1 hybrids obtained from wild *B. juncea* × glyphosate- or glufosinate-tolerant transgenic oilseed rape. Progenitors in front of the × are always maternal plants, and progenitors after the × are always paternal plants.

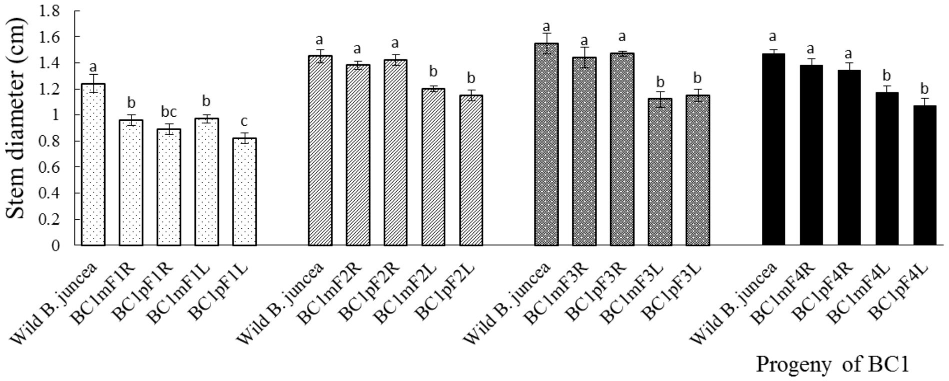

FIGURE S2-2 Stem diameter of the first to the fourth generation progenies of BC1

Data were shown as the mean ± SE. Different letters in the different progenies of the same generation indicate significant differences using Duncan's multiple range test, $P < 0.05$. BC1mF1R to BC1mF4R and BC1pF1R to BC1pF4R are the glyphosate-tolerant first to fourth generation progenies of the first backcross generation (BC1) obtained from wild *Brassica juncea* × F1R or F1R × wild *B. juncea*, respectively. BC1mF1L to BC1mF4L and BC1pF1L to BC1pF4L are the glufosinate-tolerant first to fourth generation progenies of the first backcross generation (BC1) obtained from wild *Brassica juncea* × F1L or F1L × wild *B. juncea*, respectively. F1R and F1L indicate glyphosate- or glufosinate-tolerant F1 hybrids obtained from wild *B. juncea* × glyphosate- or glufosinate-tolerant transgenic oilseed rape. Progenitors in front of the × are always maternal plants, and progenitors after the × are always paternal plants.

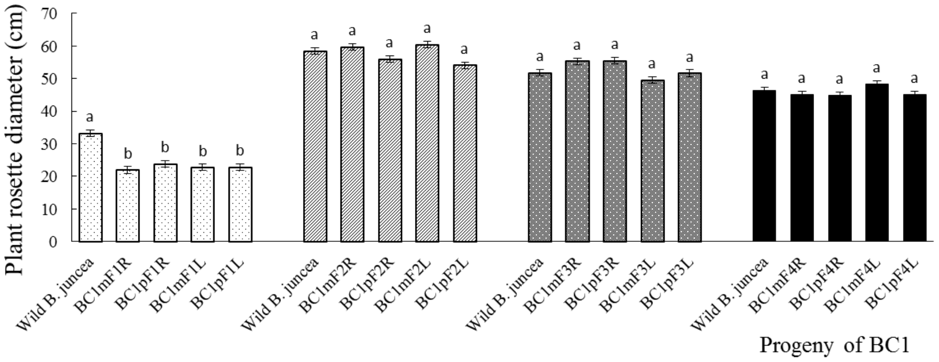

FIGURE S2-3 Plant rosette diameter of the first to the fourth generation progenies of BC1

Data were shown as the mean ± SE. Different letters in the different progenies of the same generation indicate significant differences using Duncan's multiple range test, $P < 0.05$. BC1mF1R to BC1mF4R and BC1pF1R to BC1pF4R are the glyphosate-tolerant first to fourth generation progenies of the first backcross generation (BC1) obtained from wild *Brassica juncea* × F1R or F1R × wild *B. juncea*, respectively. BC1mF1L to BC1mF4L and BC1pF1L to BC1pF4L are the glufosinate-tolerant first to fourth generation progenies of the first backcross generation (BC1) obtained from wild *Brassica juncea* × F1L or F1L × wild *B. juncea*, respectively. F1R and F1L indicate glyphosate- or glufosinate-tolerant F1 hybrids obtained from wild *B. juncea* × glyphosate- or glufosinate-tolerant transgenic oilseed rape. Progenitors in front of the × are always maternal plants, and progenitors after the × are always paternal plants.

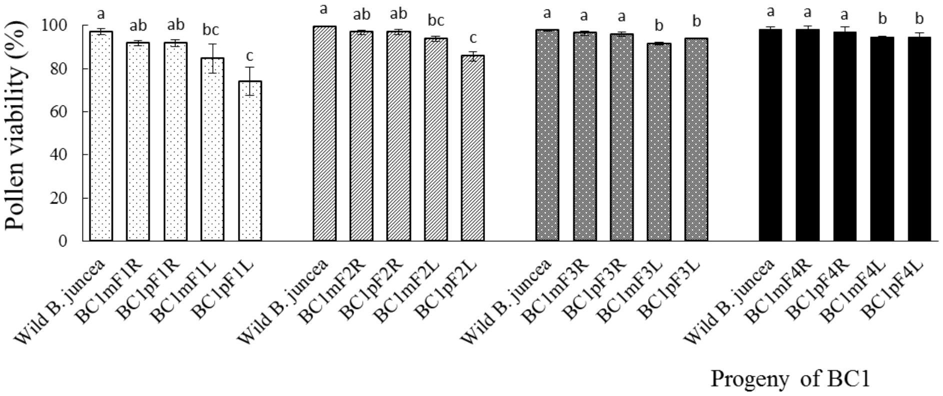

FIGURE S2-4 Pollen viability of the first to the fourth generation progenies of BC1

Data were shown as the mean ± SE. Different letters in the different progenies of the same generation indicate significant differences using Duncan's multiple range test, $P < 0.05$. BC1mF1R to BC1mF4R and BC1pF1R to BC1pF4R are the glyphosate-tolerant first to fourth generation progenies of the first backcross generation (BC1) obtained from wild *Brassica juncea* × F1R or F1R × wild *B. juncea*, respectively. BC1mF1L to BC1mF4L and BC1pF1L to BC1pF4L are the glufosinate-tolerant first to fourth generation progenies of the first backcross generation (BC1) obtained from wild *Brassica juncea* × F1L or F1L × wild *B. juncea*, respectively. F1R and F1L indicate glyphosate- or glufosinate-tolerant F1 hybrids obtained from wild *B. juncea* × glyphosate- or glufosinate-tolerant transgenic oilseed rape. Progenitors in front of the × are always maternal plants, and progenitors after the × are always paternal plants.

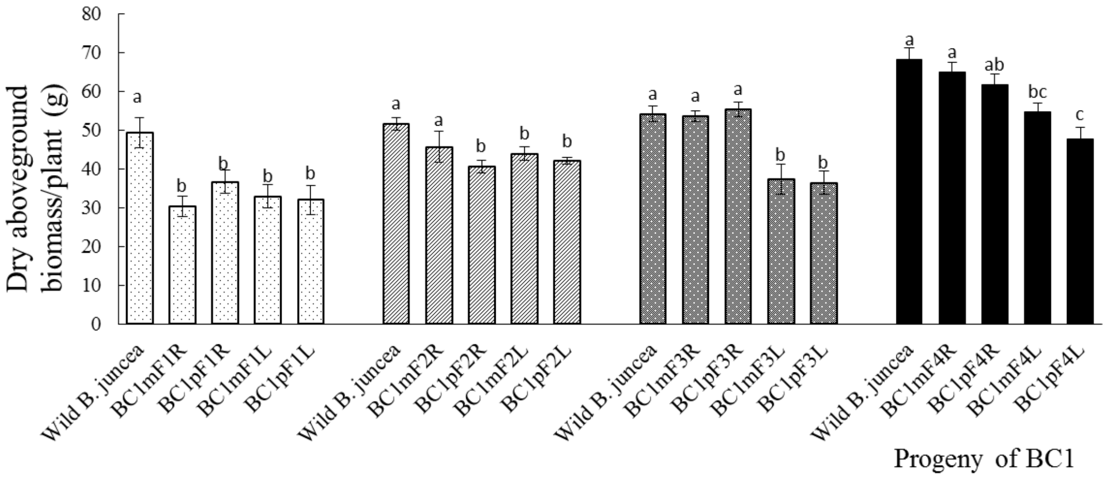

FIGURE S2-5 Dry aboveground biomass per plant of the first to the fourth generation progenies of BC1

Data were shown as the mean ± SE. Different letters in the different progenies of the same generation indicate significant differences using Duncan's multiple range test, $P < 0.05$. BC1mF1R to BC1mF4R and BC1pF1R to BC1pF4R are the glyphosate-tolerant first to fourth generation progenies of the first backcross generation (BC1) obtained from wild *Brassica juncea* × F1R or F1R × wild *B. juncea*, respectively. BC1mF1L to BC1mF4L and BC1pF1L to BC1pF4L are the glufosinate-tolerant first to fourth generation progenies of the first backcross generation (BC1) obtained from wild *Brassica juncea* × F1L or F1L × wild *B. juncea*, respectively. F1R and F1L indicate glyphosate- or glufosinate-tolerant F1 hybrids obtained from wild *B. juncea* × glyphosate- or glufosinate-tolerant transgenic oilseed rape. Progenitors in front of the × are always maternal plants, and progenitors after the × are always paternal plants.

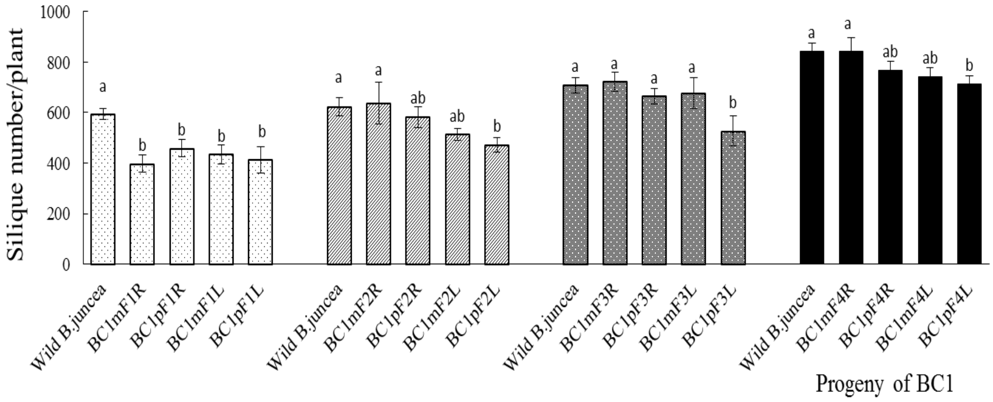

FIGURE S2-6 Silique number per plant of the first to the fourth generation progenies of BC1

Data were shown as the mean ± SE. Different letters in the different progenies of the same generation indicate significant differences using Duncan's multiple range test, $P < 0.05$. BC1mF1R to BC1mF4R and BC1pF1R to BC1pF4R are the glyphosate-tolerant first to fourth generation progenies of the first backcross generation (BC1) obtained from wild *Brassica juncea* × F1R or F1R × wild *B. juncea*, respectively. BC1mF1L to BC1mF4L and BC1pF1L to BC1pF4L are the glufosinate-tolerant first to fourth generation progenies of the first backcross generation (BC1) obtained from wild *Brassica juncea* × F1L or F1L × wild *B. juncea*, respectively. F1R and F1L indicate glyphosate- or glufosinate-tolerant F1 hybrids obtained from wild *B. juncea* × glyphosate- or glufosinate-tolerant transgenic oilseed rape. Progenitors in front of the × are always maternal plants, and progenitors after the × are always paternal plants.

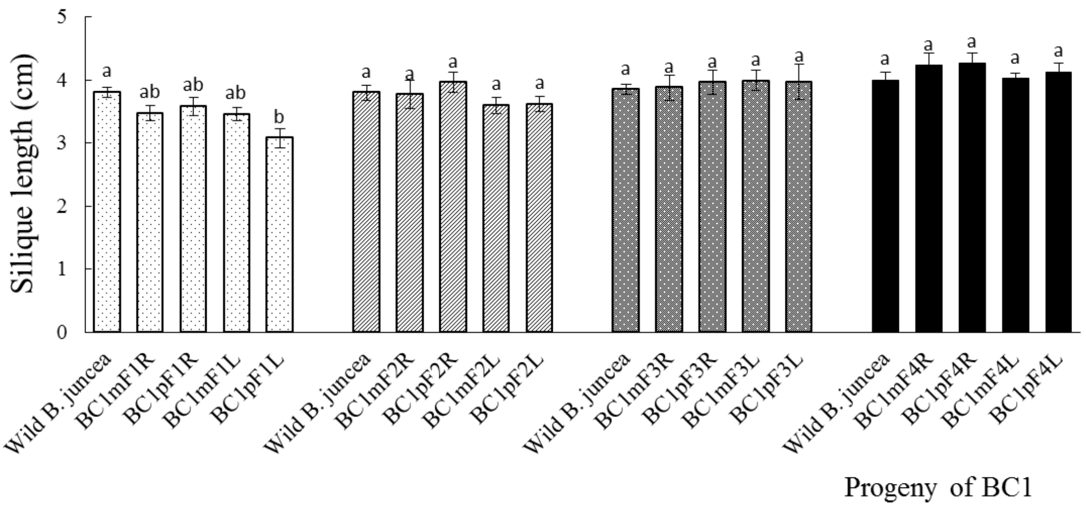

FIGURE S2-7 Silique length of the first to the fourth generation progenies of BC1

Data were shown as the mean ± SE. Different letters in the different progenies of the same generation indicate significant differences using Duncan's multiple range test, $P < 0.05$. BC1mF1R to BC1mF4R and BC1pF1R to BC1pF4R are the glyphosate-tolerant first to fourth generation progenies of the first backcross generation (BC1) obtained from wild *Brassica juncea* × F1R or F1R × wild *B. juncea*, respectively. BC1mF1L to BC1mF4L and BC1pF1L to BC1pF4L are the glufosinate-tolerant first to fourth generation progenies of the first backcross generation (BC1) obtained from wild *Brassica juncea* × F1L or F1L × wild *B. juncea*, respectively. F1R and F1L indicate glyphosate- or glufosinate-tolerant F1 hybrids obtained from wild *B. juncea* × glyphosate- or glufosinate-tolerant transgenic oilseed rape. Progenitors in front of the × are always maternal plants, and progenitors after the × are always paternal plants.

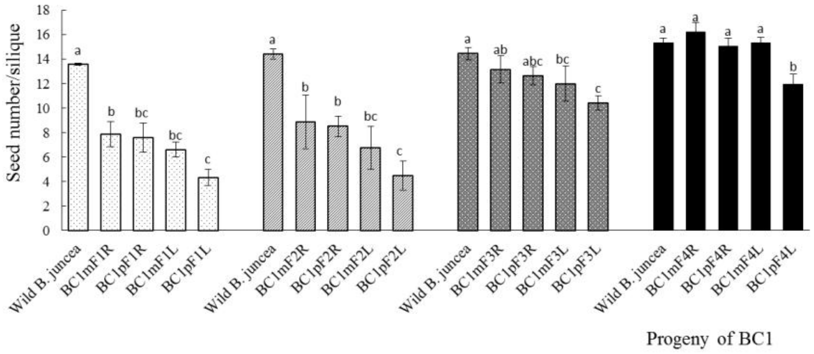

FIGURE S2-8 Seed number per silique of the first to the fourth generation progenies of BC1

Data were shown as the mean ± SE. Different letters in the different progenies of the same generation indicate significant differences using Duncan's multiple range test, $P < 0.05$. BC1mF1R to BC1mF4R and BC1pF1R to BC1pF4R are the glyphosate-tolerant first to fourth generation progenies of the first backcross generation (BC1) obtained from wild *Brassica juncea* × F1R or F1R × wild *B. juncea*, respectively. BC1mF1L to BC1mF4L and BC1pF1L to BC1pF4L are the glufosinate-tolerant first to fourth generation progenies of the first backcross generation (BC1) obtained from wild *Brassica juncea* × F1L or F1L × wild *B. juncea*, respectively. F1R and F1L indicate glyphosate- or glufosinate-tolerant F1 hybrids obtained from wild *B. juncea* × glyphosate- or glufosinate-tolerant transgenic oilseed rape. Progenitors in front of the × are always maternal plants, and progenitors after the × are always paternal plants.
