# Supplementary material for: Gene Flow Risks From Transgenic Herbicide-Tolerant Crops to Their Wild Relatives Can Be Mitigated by Utilizing Alien Chromosomes
Source: Front Plant Sci. 2021 Jun 11;12:670209. doi: 10.3389/fpls.2021.670209 (PMC8231706; doi:10.3389/fpls.2021.670209)
Supplement: Supplementary file 1 [file Data_Sheet_1.zip › Supplementary Figure S3.pdf]

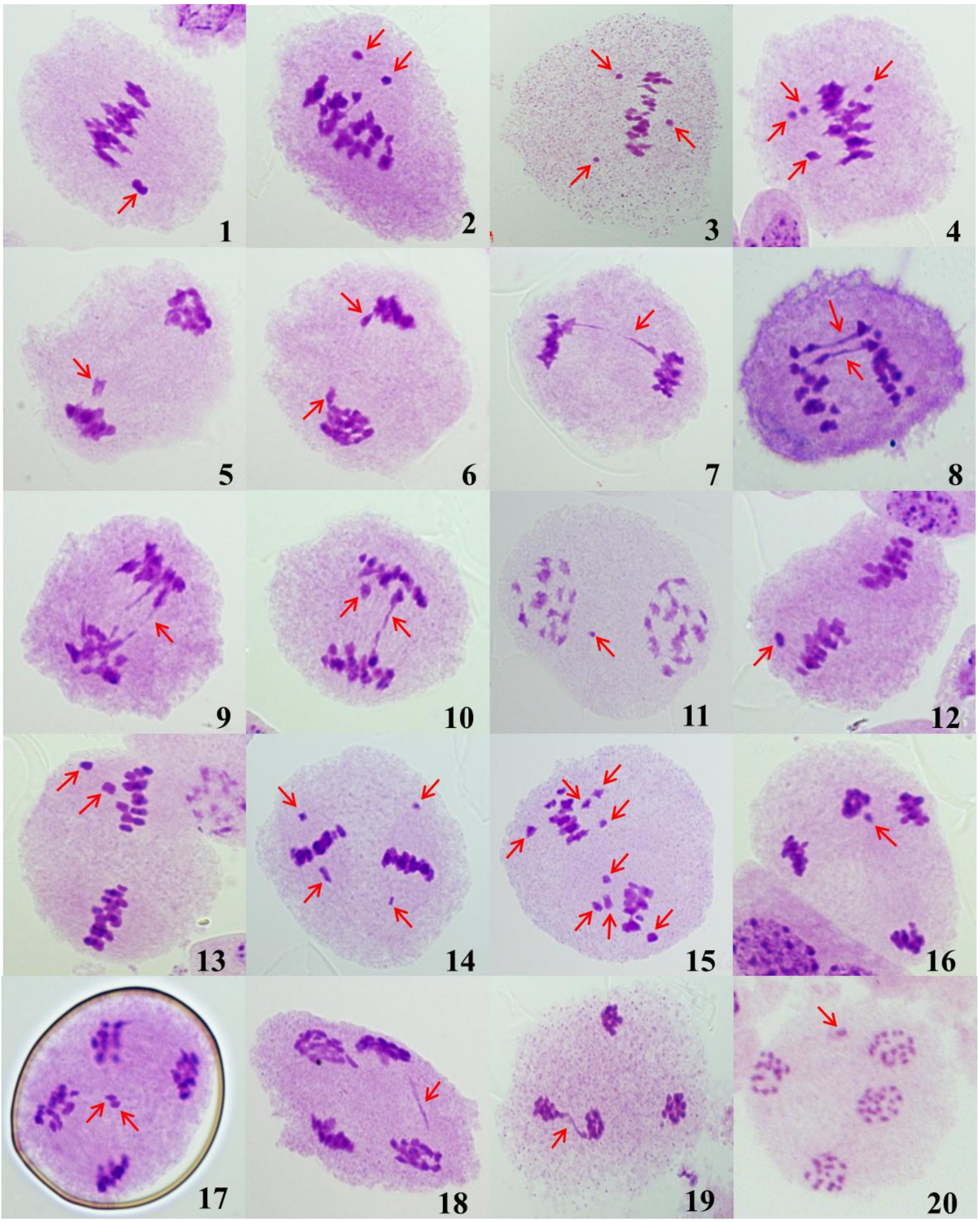

FIGURE S3-1 Microscopy observation of abnormal meiosis of pollen mother cells in BC1pF4R

Images were taken using a light microscope (ZEISS, imager. M2, 100 ×). **1-4. Methaphase Ⅰ:** 1. One lagging chromosome; 2. Two lagging chromosomes; 3. Three lagging chromosomes ; 4. Four lagging chromosomes. **5-10. Anaphase Ⅰ:** 5. One chromatid bridge; 6. Two lagging chromosomes; 7. One chromatid bridge; 8. Two chromatid bridges; 9. Double bridges; 10. One lagging chromosome and one

chromatid bridge.**11. Telophase Ⅰ:** 11. One micronucleus. **12-15. Methaphase Ⅱ:** 12. One lagging chromosome; 13. Two lagging chromosomes; 14. Four lagging chromosomes; 15. Eight lagging chromosomes. **16-19. Anaphase Ⅱ:** 16. One lagging chromosome; 17. Two lagging chromosomes; 18. One chromatid bridge; 19. One chromatid bridge. **20. Telophase Ⅱ:**20. One micronucleus.

BC1pF4R indicates the glyphosate-tolerant fourth generation progeny of the first backcross generation (BC1) obtained from F1R × wild *B. juncea*. F1R indicates glyphosate-tolerant F1 hybrids obtained from wild *B. juncea* × glyphosate-tolerant transgenic oilseed rape. Progenitors in front of the × are always maternal plants, and progenitors after the × are always paternal plants. Red arrows are indicating the abnormal meiosis of pollen mother cells.

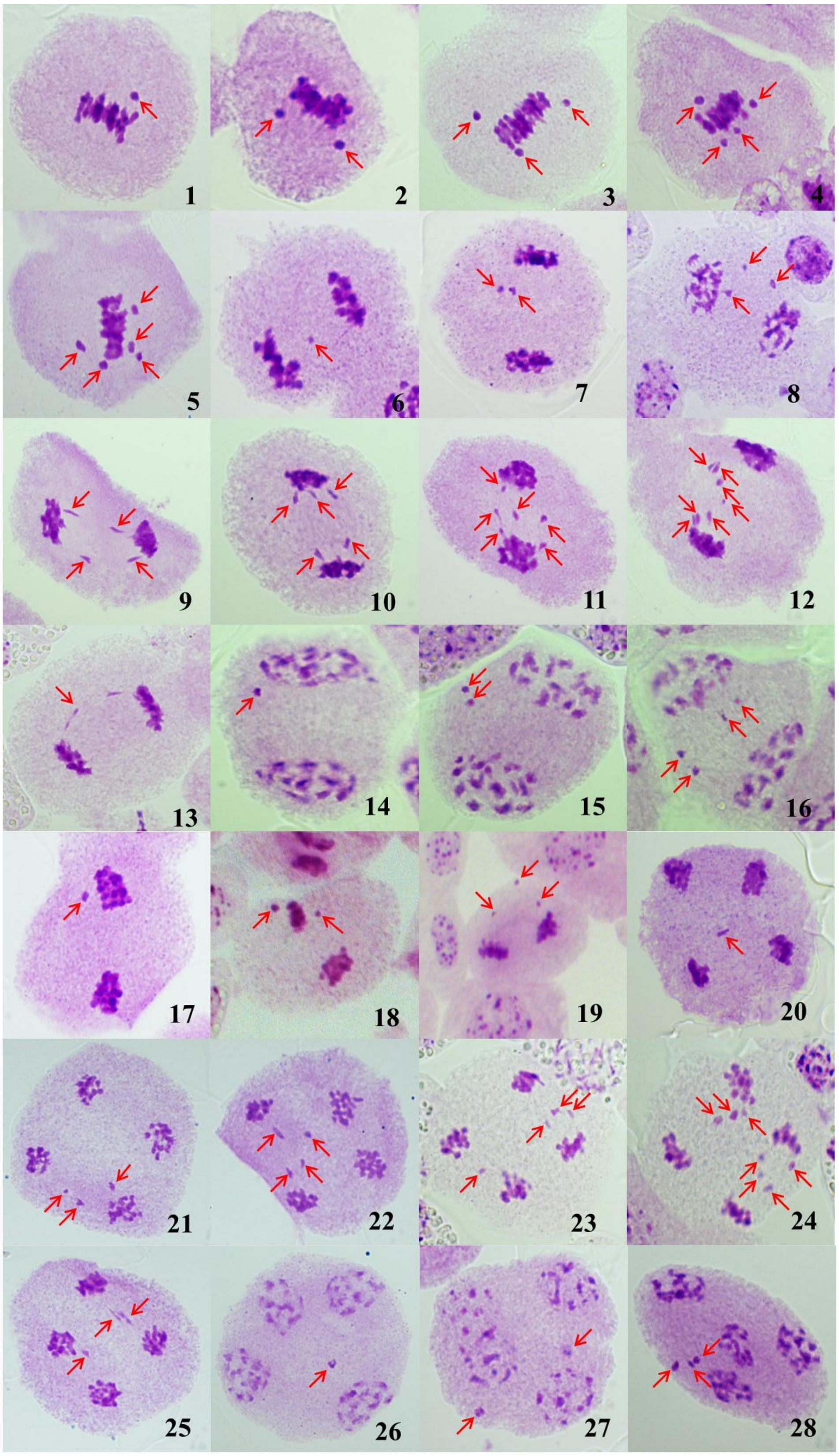

FIGURE S3-2 Microscopy observation of abnormal meiosis of pollen mother cells in BC1mF4L

Images were taken using a light microscope (ZEISS, imager. M2, 100 ×). **1-5. Methaphase Ⅰ：** 1. One

lagging chromosome; 2.Two lagging chromosomes; 3. Three lagging chromosomes; 4. Four lagging chromosomes; 5. Five lagging chromosomes. **6-13. Anaphase Ⅰ:** 6. One lagging chromosome; 7. Two lagging chromosomes; 8. Three lagging chromosomes; 9. Four lagging chromosomes; 10. Five lagging chromosomes; 11. Six lagging chromosomes; 12. Seven lagging chromosomes; 13. One chromatid bridge. **14-16. Telophase Ⅰ:** 14. One micronucleus; 15. Two micronuclei; 16. Four micronuclei. **17-19. Methaphase Ⅱ:** 17. One lagging chromosome; 18. Two lagging chromosomes; 19. Three lagging chromosomes. **20-25. Anaphase Ⅱ:** 20. One lagging chromosome; 21. Three lagging chromosomes; 22. Four lagging chromosomes; 23. Four lagging chromosomes; 24. Seven lagging chromosomes; 25. One lagging chromosome and two chromatid bridges. **26-28.Telophase Ⅱ:** 26. One micronucleus; 27. Two micronuclei; 28. Three micronuclei.

BC1mF4L indicates the glufosinate-tolerant fourth generation progeny of the first backcross generation (BC1) obtained from wild *Brassica juncea* × F1L. F1L indicates glufosinate-tolerant F1 hybrids obtained from wild *B. juncea* × glufosinate-tolerant transgenic oilseed rape. Progenitors in front of the × are always maternal plants, and progenitors after the × are always paternal plants. Red arrows are indicating the abnormal meiosis of pollen mother cells.

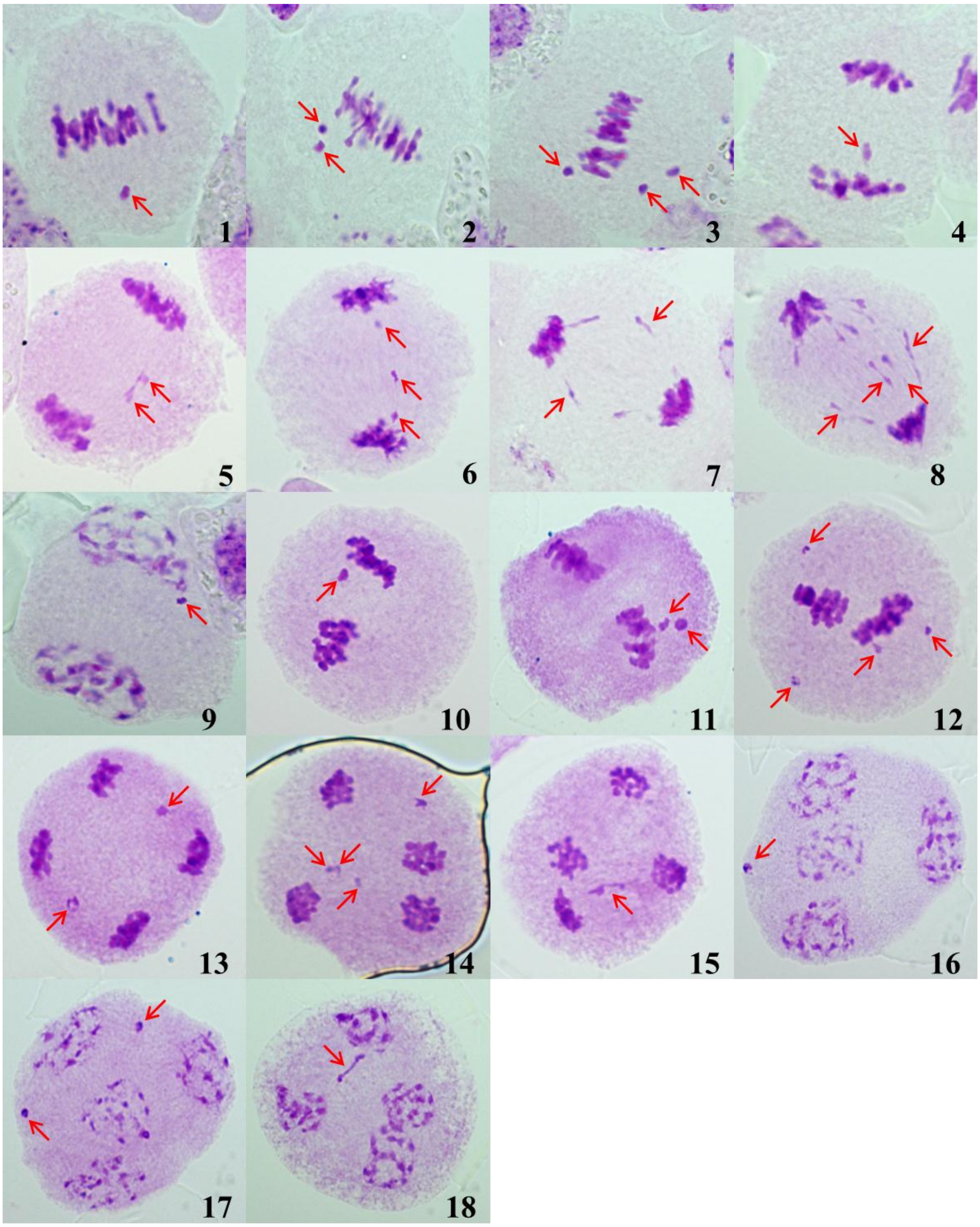

FIGURE S3-3 Microscopy observation of abnormal meiosis of pollen mother cells in BC1pF4L

Images were taken using a light microscope (ZEISS, imager. M2, 100 ×). **1-3. Methaphase Ⅰ:**1. One lagging chromosome; 2. Two lagging chromosomes; 3. Three lagging chromosomes. **4-8. Anaphase Ⅰ:** 4. One lagging chromosome; 5. Two lagging chromosomes; 6. Three lagging chromosomes; 7. Two

chromatid bridges; 8. Multiple lagging chromosomes and chromatid bridges. **9. Telophase Ⅰ:** 9. One micronucleus. **10-12. Methaphase Ⅱ:** 10. One lagging chromosome; 11. Two lagging chromosomes; 12. Four lagging chromosomes. **13-15. Anaphase Ⅱ:** 13. Two lagging chromosomes; 14. Four lagging chromosomes; 15. One chromatid bridge. **16-18. Telophase Ⅱ:**16. One micronucleus; 17. Two micronuclei; 18. One chromatid bridge.

BC1pF4L indicates the glufosinate-tolerant fourth generation progeny of the first backcross generation (BC1) obtained from × wild *B. juncea*. F1L indicates glufosinate-tolerant F1 hybrids obtained from *B. juncea* × glufosinate-tolerant transgenic oilseed rape. Progenitors in front of the × are always maternal plants, and progenitors after the × are always paternal plants. Red arrows are indicating the abnormal meiosis of pollen mother cells.
