# Supplementary material for: Gene Flow Risks From Transgenic Herbicide-Tolerant Crops to Their Wild Relatives Can Be Mitigated by Utilizing Alien Chromosomes
Source: Front Plant Sci. 2021 Jun 11;12:670209. doi: 10.3389/fpls.2021.670209 (PMC8231706; doi:10.3389/fpls.2021.670209)
Supplement: Supplementary file 1 [file Data_Sheet_1.zip › Supplementary Table S1.pdf]

TABLE S1 Traits measured for relative fitness estimation at two life cycle stages

	Traits	Methods
Vegetative	Plant height	Height from the base of the plant to the tip of the plant at maturity.
	Stem diameter	Main stem diameter of each plant measured at maturity.
	plant rosette diameter	Maximum diameter of plant rosette measured at bolting stage.
	Dry above-biomass	Measured at final harvest as described by Liu et al. (2010).
Reproductive	Pollen viability	Assessed as the percentage of pollen stained by a 1% aceto-carmine solution in a sample of 30 plants in each generation progeny (3 plants from each plant lineage, 3 flowers and at least 600 pollen grains for each plant).
	Number of silique / plant	Number of siliques / plant at maturity.
	Silique length	Silique length at lower part of plant measured at maturity stage (sample of at least 20 siliques per plant).
	Seed number/silique	Number of filled seeds in silique at measuring silique length.
