# Supplementary material for: Gene Flow Risks From Transgenic Herbicide-Tolerant Crops to Their Wild Relatives Can Be Mitigated by Utilizing Alien Chromosomes
Source: Front Plant Sci. 2021 Jun 11;12:670209. doi: 10.3389/fpls.2021.670209 (PMC8231706; doi:10.3389/fpls.2021.670209)
Supplement: Supplementary file 1 [file Data_Sheet_1.zip › Supplementary Table S2.pdf]

TABLE S2 Sixteen markers specific to the C-chromosome found in *Brassica napus* but completely absent in *B. rapa*, *B. nigra*, and *B. juncea*

N	PN	C	AL	FP	RP
1	CB10026	C2	136	TCGTTCTGACCTGTCGTTAT	GGAAATGGCTGCTCATGTT
2	Ol13-G05	C2	145	GTGTGCAGGAAACGATGTTC	GGGAGTTTGAAGAGAAAGCG
3	Na10-D03	C3	181	ATGATTTGCCTTGAAATGCC	GATGAAACAATAACCTGAGACACAC
4	BRAS087	C3	181	GCAGAATAGCCTCGCA	GGAGGAAAAGAACGTGG
5	CB10057	C3	190	CTAGGCTAAGGAAGATTGTCA	TAGTTTCTTCCTCCTGCTATC
6	CB10132	C3	244	CCTGTGGAGACCGTGACTACA	AATTTCGACACAACTGCTTAG
7	Na12-G04	C4	183	CGAATTGAAGGATGAGTTTGG	CACATGTTTTATCATTCACAAGTCC
8	MR129	C5	302	CGGGTTGTCAATGAATAAGTA	AACACCCCCGATACACTAA
9	CB10234	C6	320	TCTGTTGTTTCTCTCGCC	CTGATGGACTAGGACCCC
10	CB10502	C6	295	TTGAAGAGTGGGGATTCA	GGTGAGCTTCTTCCTTCC
11	Ra2-A05	C6	64	GCTAGTTTACGCGGCGG	AAACGACATCGGCAAAGAAG
12	CB10297	C7	276	CTCATTCCACTGCCAAAC	ACGAGTCACCATGTCAGG
13	CB10528	C7	297	ATGCTTTCTTTGCACGAG	ACCAGACTGATGGTGTGC
14	BRAS019	C7	148	CTCAAGACAAACGACCAGTAA	GAGAAGAAATCGCCAAGA
15	Ol12-G04	C8	122	CGAACATCTTAGGCCGAATC	GGTTAACCTGCGGGATATTG
16	CB10139	C8	170	TCTCAAAAGGATATGCGTGAA	CAAAACTCATCAGGGTTGTAG

N means Number. PN means primer name. C means chromosome. AL means application length. FP means forward primer. RP means reverse primer.
