# Supplementary material for: Gene Flow Risks From Transgenic Herbicide-Tolerant Crops to Their Wild Relatives Can Be Mitigated by Utilizing Alien Chromosomes
Source: Front Plant Sci. 2021 Jun 11;12:670209. doi: 10.3389/fpls.2021.670209 (PMC8231706; doi:10.3389/fpls.2021.670209)
Supplement: Supplementary file 1 [file Data_Sheet_1.zip › Supplementary Table S3.pdf]

TABLE S3-1 Surviving number and percentage of the first to fourth generation progenies of BC1 after spraying with glyphosate

Progeny	observed		Percentage of surviving plants (%)	X^2 value under expected ratio	Expected ratio
	Number of surviving plants	Number of dead plants			
BC1mF1(R)	1311	480	73.20	3.00	3:1
BC1pF1(R)	1215	450	72.97	3.54	3:1
BC1mF2(R)	832	190	81.41	2.59	5:1
BC1pF2(R)	827	192	81.16	3.32	5:1
BC1mF3(R)	1520	166	90.15	0.03	9:1
BC1pF3(R)	896	112	88.89	1.26	9:1
BC1mF4(R)	1109	49	95.77	3.41	17:1
BC1pF4(R)	1092	81	93.09	3.60	17:1

BC1mF1(R) to BC1mF4(R) and BC1pF1(R) to BC1pF4(R) are the first to fourth generation progenies of the first backcross generation (BC1) obtained from wild *Brassica juncea* × F1R or F1R × wild *B. juncea*, respectively. F1R indicates glyphosate-tolerant F1 hybrids obtained from wild *B. juncea* × glyphosate-tolerant transgenic oilseed rape. Progenitors in front of the × are always maternal plants, and progenitors after the × are always paternal plants. The dosage of glyphosate used was 1037 g (a.i.) ha^{-1}.

TABLE S3-2 Surviving number and percentage of the first to fourth generation progenies of BC1 after spraying with glufosinate

Progeny	observed		Percentage of surviving plants (%)	X^2value under expected ratio	Expected ratio
	Number of surviving plants	Number of dead plants			
BC1mF1(L)	902	882	50.56	567.00*	3:1
BC1pF1(L)	698	591	54.15	297.73*	3:1
BC1mF2(L)	1409	1379	50.54	2156.62*	5:1
BC1pF2(L)	1285	1088	54.15	1452.94*	5:1
BC1mF3(L)	709	586	54.75	1784.09*	9:1
BC1pF3(L)	502	382	56.79	1079.78*	9:1
BC1mF4(L)	709	586	54.75	1784.09*	17:1
BC1pF4(L)	726	643	53.03	4466.91*	17:1

*Significant deviation ($P < 0.05$) from expected ratio.

BC1mF1(L) to BC1mF4(L) and BC1pF1(L) to BC1pF4(L) are the first to fourth generation progenies of the first backcross generation (BC1) obtained from wild *Brassica juncea* × F1L or F1L × wild *B. juncea*, respectively. F1L indicates the glufosinate -tolerant F1 hybrids obtained from wild *B. juncea* ×glufosinate-tolerant transgenic oilseed rape. Progenitors in front of the × are always maternal plants, and progenitors after the × are always paternal plants. The dosage of glufosinate used was 700 g (a.i.) ha^{-1}.
