# Supplementary material for: Gene Flow Risks From Transgenic Herbicide-Tolerant Crops to Their Wild Relatives Can Be Mitigated by Utilizing Alien Chromosomes
Source: Front Plant Sci. 2021 Jun 11;12:670209. doi: 10.3389/fpls.2021.670209 (PMC8231706; doi:10.3389/fpls.2021.670209)
Supplement: Supplementary file 1 [file Data_Sheet_1.zip › Supplementary Table S4.pdf]

TABLE S4-1 Percentage of ovary cells with different chromosomes in BC1mF1R

Plant	RCN	chromosome number									
		＜35	35	36	37	38	39	40	41	42	＞45
1	32-37	18.75	9.38	21.88	50.00	0	0	0	0	0	0
2	35-＞45	0	14.81	37.04	18.52	22.22	0	0	0	0	7.41
3	30-＞45	9.52	9.52	69.05	0	0	0	0	0	0	11.90
4	32-＞45	15.38	0	3.85	7.69	69.23	0	0	0	0	3.85
5	29-＞45	6.67	0	0	3.33	16.67	23.33	36.67	10.00	0	3.33
6	28-＞45	13.33	0	3.33	6.67	10.00	36.67	16.67	0	0	13.33
7	35-41	0	3.57	3.57	3.57	14.29	3.57	67.86	3.57	0	0
8	36-42	0	0	3.70	0	3.70	18.52	25.93	40.74	7.41	0
9	31-＞45	8.70	0	8.70	8.70	26.09	43.48	0	0	0	4.35

RCN means range of chromosome number. At least 30 cells from each of plant were analyzed.

BC1mF1R indicates the glyphosate-tolerant first generation progeny of the first backcross generation (BC1) obtained from wild *Brassica juncea* × F1R. F1R indicates the glyphosate-tolerant F1 hybrids obtained from wild *B. juncea* × glyphosate-tolerant transgenic oilseed rape. Progenitors in front of the × are always maternal plants, and progenitors after the × are always paternal plants..

TABLE S4-2 Percentage of ovary cells with different chromosomes in BC1pF1R

Plant	RCN	chromosome number											
		＜35	35	36	37	38	39	40	41	42	43	44	＞45
1	30-37	16.00	16.00	12.00	56.00	0	0	0	0	0	0	0	0
2	27-＞45	26.83	24.39	26.83	7.32	9.76	2.44	0	0	0	0	0	2.44
3	35-＞45	0	3.57	10.71	28.57	10.71	39.29	0	0	0	0	0	7.14
4	27-42	6.82	2.27	2.27	2.27	13.64	11.36	45.45	13.64	2.27	0	0	0
5	28-＞45	4.00	4.00	8.00	64.00	8.00	8.00	0	0	0	0	0	4.00
6	34-40	8.33	4.17	8.33	4.17	4.17	4.17	66.67	0	0	0	0	0
7	35-＞45	0	3.57	3.57	3.57	0	0	7.14	7.14	0	50.00	3.57	21.43
8	33-＞45	8.33	70.83	16.67	0	0	0	0	0	0	0	0	4.17
9	32-38	10.00	0	15.00	5.00	70.00	0	0	0	0	0	0	0
10	≥51	0	0	0	0	0	0	0	0	0	0	0	100

RCN means range of chromosome number. At least 30 cells from each of plant were analyzed.

BC1pF1R indicates the glyphosate-tolerant first generation progeny of the first backcross generation (BC1) obtained from F1R × wild *B. juncea.* F1R indicates the glyphosate-tolerant F1 hybrids obtained from wild *B. juncea* × glyphosate-tolerant transgenic oilseed rape. Progenitors in front of the × are always maternal plants, and progenitors after the × are always paternal plants.

TABLE S4-3 Percentage of ovary cells with different chromosomes in BC1mF2R

Plant	RCN	chromosome number				
		＜35	35	36	37	＞45
1	26-37	31.25	6.25	59.38	3.13	0
2	30-＞45	10.00	16.67	33.33	0	40.00
3	28-36	32.26	51.61	16.13	0	0
4	29-36	29.03	9.68	61.29	0	0
5	26-＞45	26.67	13.33	56.67	0	3.33
6	26-36	31.25	43.75	25.00	0	0
7	34-36	5.00	10.00	85.00	0	0
8	29-37	35.71	7.14	42.86	14.29	0

RCN means range of chromosome number. At least 30 cells from each of plant were analyzed. BC1mF2R indicates the glyphosate-tolerant second generation progeny of the first backcross generation (BC1) obtained from wild *Brassica juncea* × F1R. F1R indicates the glyphosate-tolerant F1 hybrids obtained from wild *B. juncea* × glyphosate-tolerant transgenic oilseed rape. Progenitors in front of the × are always maternal plants, and progenitors after the × are always paternal plants.

TABLE S4-4 Percentage of ovary cells with different chromosomes in BC1pF2R

Plant	Range of Chromosome number	chromosome number							
		＜35	35	36	37	38	39	40	＞45
1	30-39	32.56	6.98	20.93	37.21	0	2.33	0	0
2	28-38	16.22	0	24.32	54.05	5.41	0	0	0
3	28-38	33.33	3.03	60.61	0	3.03	0	0	0
4	26-38	23.33	16.67	53.33	0	6.67	0	0	0
5	26-40	28.00	0	12.00	20.00	32.00	4.00	4.00	0
6	29-＞45	14.29	9.52	52.38	14.29	4.76	0	0	4.76
7	34-38	8.70	0	26.09	4.35	60.87	0	0	0
8	33-36	23.81	9.52	66.67	0	0	0	0	0

RCN means range of chromosome number. At least 30 cells from each of plant were analyzed.

BC1pF2R indicates the glyphosate-tolerant second generation progeny of the first backcross generation (BC1) obtained from F1R × wild *Brassica juncea*. F1R indicates the glyphosate-tolerant F1 hybrids obtained from wild *B. juncea* × glyphosate-tolerant transgenic oilseed rape. Progenitors in front of the × are always maternal plants, and progenitors after the × are always paternal plants.

TABLE S4-5 Percentage of ovary cells with different chromosomes in BC1mF3R

Plant	RCN	chromosome number			
		<35	35	36	37
1	28-36	18.52	11.11	70.37	0
2	29-36	26.92	7.69	65.38	0
3	29-36	21.74	8.70	69.57	0
4	26-36	28.57	19.05	52.38	0
5	32-36	26.67	13.33	60.00	0
6	28-36	26.67	6.67	66.67	0
7	28-37	23.08	7.69	61.54	7.69

RCN means range of chromosome number. At least 30 cells from each of plant were analyzed.

BC1mF3R indicates the glyphosate-tolerant third generation progeny of the first backcross generation (BC1) obtained from wild *Brassica juncea* × F1R. F1R indicates the glyphosate-tolerant F1 hybrids obtained from wild *B. juncea* × glyphosate-tolerant transgenic oilseed rape. Progenitors in front of the × are always maternal plants, and progenitors after the × are always paternal plants.

TABLE S4-6 Percentage of ovary cells with different chromosomes in BC1pF3R

Plant	RCN	chromosome number						
		＜35	35	36	37	38	39	＞45
1	26-＞45	20.93	32.56	44.19	0	0	0	2.33
2	32-38	19.23	7.69	65.38	3.85	3.85	0	0
3	33-37	16.67	8.33	70.83	4.17	0	0	0
4	31-36	19.05	0.00	80.95	0	0	0	0
5	28-36	26.92	15.38	57.69	0	0	0	0
6	27-36	20.83	12.50	66.67	0	0	0	0
7	29-36	35.00	5.00	60.00	0	0	0	0
8	28-36	20.00	10.00	70.00	0	0	0	0

RCN means range of chromosome number. At least 30 cells from each of plant were analyzed. BC1pF3R indicates the glyphosate-tolerant third generation progeny of the first backcross generation (BC1) obtained from F1R × wild *B. juncea.* F1R indicates the glyphosate-tolerant F1 hybrids obtained from wild *B. juncea* × glyphosate-tolerant transgenic oilseed rape. Progenitors in front of the × are always maternal plants, and progenitors after the × are always paternal plants.

TABLE S4-7 Percentage of ovary cells with different chromosomes in BC1mF4R

Plant	RCN	chromosome number						
		＜35	35	36	37	38	41	＞45
1	31-38	11.54	1.92	5.77	23.08	57.69	0	0
2	31-41	23.08	10.26	64.10	0	0	2.56	0
3	30-36	20.00	10.00	70.00	0	0	0	0
4	31-＞45	29.17	33.33	33.33	0	0	0	4.17
5	28-36	13.33	16.67	70.00	0	0	0	0
6	31-36	27.78	2.78	69.44	0	0	0	0
7	28-38	27.78	19.44	47.22	2.78	2.78	0	0
8	29-＞45	20.59	8.82	61.76	0	0	0	8.82

RCN means range of chromosome number. At least 30 cells from each of plant were analyzed. BC1mF4R indicates the glyphosate-tolerant fourth generation progeny of the first backcross generation (BC1) obtained from wild *Brassica juncea* × F1R. F1R indicates the glyphosate-tolerant F1 hybrids obtained from wild *B. juncea* × glyphosate-tolerant transgenic oilseed rape. Progenitors in front of the × are always maternal plants, and progenitors after the × are always paternal plants.

TABLE S4-8 Percentage of ovary cells with different chromosomes in BC1pF4R

Plant	RCN	chromosome number						
		＜35	35	36	37	38	44	＞45
1	26-36	41.86	55.81	2.33	0	0	0	0
2	30-38	18.42	7.89	71.05	0	2.63	0	0
3	32-37	12.12	9.09	75.76	3.03	0	0	0
4	34-＞44	3.23	12.90	35.48	45.16	0	0	3.23
5	23-＞44	22.86	8.57	60.00	2.86	0	0	5.71
6	22-＞44	44.19	25.58	23.26	0	0	0	6.98
7	29-＞44	18.75	9.38	65.63	0	0	0	6.25
8	31-44	22.58	25.81	45.16	0	0	3.23	3.23

RCN means range of chromosome number. At least 30 cells from each of plant were analyzed. BC1pF4R indicates the glyphosate-tolerant fourth generation progeny of the first backcross generation (BC1) obtained from F1R × wild *B. juncea*. F1R indicates the glyphosate-tolerant F1 hybrids obtained from wild *B. juncea* × glyphosate-tolerant transgenic oilseed rape. Progenitors in front of the × are always maternal plants, and progenitors after the × are always paternal plants.

TABLE S4-9 Percentage of ovary cells with different chromosomes in BC1mF1L

Plant	RCN	chromosome number										
		＜36	36	37	38	39	40	41	42	43	44	＞45
1	28-＞45	19.44	0	0	13.89	11.11	36.11	2.78	0	0	0	16.67
2	36-＞45	0	6.45	0	0	3.23	6.45	19.35	3.23	29.03	29.03	3.23
3	37-40	0	0	6.45	3.23	9.68	80.65	0	0	0	0	0
4	29-＞45	10.00	0	6.67	20.00	16.67	40.00	0	0	0	0	6.67
5	32-＞45	3.23	12.90	54.84	16.13	0	0	0	0	0	0	12.90
6	28-40	8.00	0	24.00	44.00	20.00	4.00	0	0	0	0	0
7	39-＞45	0	0	0	0	8.33	16.67	16.67	12.50	20.83	20.83	4.17
8	38-43	0	0	0	4.35	4.35	13.04	8.70	65.22	4.35	0	0

RCN means range of chromosome number. At least 30 cells from each of plant were analyzed.

BC1mF1L indicates the glufosinate-tolerant first generation progeny of the first backcross generation (BC1) obtained from wild *Brassica juncea* × F1L. F1L indicates the glufosinate-tolerant F1 hybrids obtained from wild *B. juncea* × glufosinate-tolerant transgenic oilseed rape. Progenitors in front of the × are always maternal plants, and progenitors after the × are always paternal plants.

TABLE S4-10 Percentage of ovary cells with different chromosomes in BC1pF1L

Plant	RCN	chromosome number									
		＜36	36	37	38	39	40	41	42	43	＞45
1	29-43	5.41	0	00	5.41	8.11	5.41	2.70	8.11	64.86	0
2	32-＞45	17.65	2.94	8.82	50.00	11.76	0	0	0	0	8.82
3	33-39	6.25	3.13	65.63	18.75	6.25	0	0	0	0	0
4	35-＞45	3.85	0	0	3.85	7.69	26.92	53.85	0	0	3.85
5	29-＞45	50.00	26.92	19.23	0	0	0	0	0	0	3.85
6	37-＞45	0	0	10.00	5.00	15.00	15.00	30.00	5.00	10.00	10.00
7	34-＞45	9.52	4.76	14.29	4.76	23.81	9.52	0	0	0	33.33
8	34-40	10.00	0	0	10.00	30.00	50.00	0	0	0	0

RCN means range of chromosome number. At least 30 cells from each of plant were analyzed.

BC1pF1L indicates the glufosinate-tolerant first generation progeny of the first backcross generation (BC1) obtained from F1L × wild *B. juncea*. F1L indicates the glufosinate-tolerant F1 hybrids obtained from wild *B. juncea* × glufosinate-tolerant transgenic oilseed rape. Progenitors in front of the × are always maternal plants, and progenitors after the × are always paternal plants.

TABLE S4-11 Percentage of ovary cells with different chromosomes in BC1mF2L

Plant	RCN	chromosome number								
		＜36	36	37	38	39	40	41	42	＞45
1	28-40	19.57	0	17.39	41.30	17.39	4.35	0	0	0
2	26-41	17.39	13.04	4.35	19.57	17.39	19.57	8.70	0	0
3	26-37	34.62	0	65.38	0	0	0	0	0	0
4	26-37	20.00	16.00	64.00	0	0	0	0	0	0
5	28-＞45	47.62	4.76	42.86	0	0	0	0	0	4.76
6	26-37	38.10	4.76	57.14	0	0	0	0	0	0
7	36-42	0	5.00	30.00	25.00	0	5.00	0	35.00	0
8	27-37	23.81	9.52	66.67	0	0	0	0	0	0

RCN means range of chromosome number. At least 30 cells from each of plant were analyzed.

BC1mF2L indicates the glufosinate-tolerant second generation progeny of the first backcross generation (BC1) obtained from wild *Brassica juncea* × F1L. F1L indicates the glufosinate-tolerant F1 hybrids obtained from wild *B. juncea* × glufosinate-tolerant transgenic oilseed rape. Progenitors in front of the × are always maternal plants, and progenitors after the × are always paternal plants.

TABLE S4-12 Percentage of ovary cells with different chromosomes in BC1pF2L

Plant	RCN	chromosome number								
		＜36	36	37	38	39	40	41	42	＞45
1	32-＞45	8.89	20.00	15.56	37.78	11.11	4.44	0	0	2.22
2	29-37	44.12	20.59	35.29	0	0	0	0	0	0
3	29-＞45	68.75	12.50	12.50	0	0	0	0	0	6.25
4	27-37	22.58	41.94	35.48	0	0	0	0	0	0
5	27-42	22.73	4.55	9.09	0	4.55	18.18	31.82	9.09	0
6	34-＞45	19.05	4.76	71.43	0	0	0	0	0	4.76
7	36-38	0	13.64	81.82	4.55	0	0	0	0	0
8	29-41	14.29	4.76	0	14.29	0	19.05	47.62	0	0

RCN means range of chromosome number. At least 30 cells from each of plant were analyzed.

BC1pF2L indicates the glufosinate-tolerant second generation progeny of the first backcross generation (BC1) obtained from F1L × wild *B. juncea.* F1L indicates the glufosinate-tolerant F1 hybrids obtained from wild *B. juncea* × glufosinate-tolerant transgenic oilseed rape. Progenitors in front of the × are always maternal plants, and progenitors after the × are always paternal plants.

TABLE S4-13 Percentage of ovary cells with different chromosomes in BC1mF3L

Plant	RCN	chromosome number				
		＜36	36	37	38	＞45
1	34-38	6.90	10.34	27.59	55.17	0
2	31-37	17.86	17.86	64.29	0	0
3	34-38	17.86	10.71	53.57	17.86	0
4	34-38	16.00	12.00	64.00	8.00	0
5	31-37	20.00	8.00	72.00	0	0
6	34-38	16.00	16.00	64.00	4.00	0
7	30-37	27.27	9.09	63.64	0	0
8	34-＞45	9.52	14.29	71.43	0	4.76
9	32-37	23.81	4.76	71.43	0	0
10	29-37	30.00	5.00	65.00	0	0

RCN means range of chromosome number. At least 30 cells from each of plant were analyzed.

BC1mF3L indicates the glufosinate-tolerant third generation progeny of the first backcross generation (BC1) obtained from wild *Brassica juncea* ×F1L. F1L indicates the glufosinate-tolerant F1 hybrids obtained from wild *B. juncea* ×glufosinate-tolerant transgenic oilseed rape. Progenitors in front of the × are always maternal plants, and progenitors after the ×are always paternal plants.

TABLE S4-14 Percentage of ovary cells with different chromosomes in BC1pF3L

Plant	RCN	chromosome number							
		＜36	36	37	38	39	40	41	＞45
1	28-41	21.05	10.53	34.21	10.53	18.42	2.63	2.63	0
2	27-40	17.14	8.57	20.00	20.00	8.57	25.71	0	0
3	33-＞45	18.75	6.25	68.75	3.13	0	0	0	3.13
4	28-＞45	19.23	11.54	65.38	0	0	0	0	3.85
5	34-38	16.00	8.00	72.00	4.00	0	0	0	0
6	29-37	26.09	21.74	52.17	0	0	0	0	0
7	31-37	27.27	9.09	63.64	0	0	0	0	0
8	26-＞45	28.57	23.81	38.10	0	0	0	0	9.52
9	27-37	23.81	14.29	61.90	0	0	0	0	0
10	36-＞45	0	9.52	80.95	0	0	0	0	9.52

RCN means range of chromosome number. At least 30 cells from each of plant were analyzed.

BC1pF3L indicates the glufosinate-tolerant third generation progeny of the first backcross generation (BC1) obtained from F1L × wild *B. juncea*. F1L indicates the glufosinate-tolerant F1 hybrids obtained by wild *B. juncea* × glufosinate-tolerant transgenic oilseed rape. Progenitors in front of the × are always maternal plants, and progenitors after the × are always paternal plants.

TABLE S4-15 Percentage of ovary cells with different chromosomes in BC1mF4L

Plant	RCN	Chromosome number				
		＜36	36	37	38	＞45
1	28-37	35.14	24.32	40.54	0	0
2	32-38	16.67	16.67	61.11	5.56	0
3	31-＞45	5.71	17.14	22.86	48.57	5.71
4	33-37	18.75	25.00	56.25	0	0
5	31-＞45	24.24	21.21	51.52	0	3.03
6	35-37	6.25	25.00	68.75	0	0
7	28-＞45	38.71	6.45	51.61	0	3.23
8	33-37	21.88	6.25	71.88	0	0

RCN means range of chromosome number. At least 30 cells from each of plant were analyzed. BC1mF4L indicates the glufosinate-tolerant fourth generation progeny of the first backcross generation (BC1) obtained from wild *Brassica juncea* ×F1L. F1L indicates the glufosinate -tolerant F1 hybrids obtained from wild *B. juncea* ×glufosinate-tolerant transgenic oilseed rape. Progenitors in front of the ×are always maternal plants, and progenitors after the ×are always paternal plants.

TABLE S4-16 Percentage of ovary cells with different chromosomes in BC1pF4L

Plant	RCN	chromosome number				
		＜36	36	37	38	＞45
1	29-＞45	30.95	21.43	45.24	0	2.38
2	32-37	25.71	17.14	57.14	0	0
3	29-37	23.33	20.00	56.67	0	0
4	30-37	20.51	7.69	71.79	0	0
5	30-38	17.39	8.70	60.87	13.04	0
6	34-38	27.27	13.64	54.55	4.55	0
7	31-38	19.05	9.52	66.67	4.76	0
8	29-38	15.00	5.00	75.00	5.00	0
9	36-37	0	35.00	65.00	0	0

RCN means range of chromosome number. At least 30 cells from each of plant were analyzed.

BC1pF4L indicates the glufosinate-tolerant fourth generation progeny of the first backcross generation (BC1) obtained from F1L × wild *B. juncea.* F1L indicates the glufosinate-tolerant F1 hybrids obtained by wild *B. juncea* × glufosinate-tolerant transgenic oilseed rape. Progenitors in front of the × are always maternal plants, and progenitors after the × are always paternal plants.
