# Supplementary material for: Gene Flow Risks From Transgenic Herbicide-Tolerant Crops to Their Wild Relatives Can Be Mitigated by Utilizing Alien Chromosomes
Source: Front Plant Sci. 2021 Jun 11;12:670209. doi: 10.3389/fpls.2021.670209 (PMC8231706; doi:10.3389/fpls.2021.670209)
Supplement: Supplementary file 1 [file Data_Sheet_1.zip › Supporting information 1.pdf]

Supporting information 1

Materials and Methods

DNA extraction and flank sequencing

Total DNA was extracted from leaves of the two kinds of herbicide tolerant transgenic oilseed rape using SDS method, and 10 milligrams of dry rape leaves were used, respectively. Flanking sequences were obtained by thermal asymmetric interlaced PCR (TAIL-PCR) as described by Liu & Whittier (1995). The gene-specific primers were designed based on the *cp4-epsps* and FMV 35S, *pat* and CaMV 35S DNA sequences, respectively. The anchor primers were selected according to Liu & Whittier (1995). The nucleotides for these primers are listed in Table 1. The reaction mixture for flanking sequences amplification was listed in Table 2 and the reaction programmes detailed in Table 3.

Cloning and sequence analysis of PCR products

PCR products were purified recovered in accordance with the Agent BioMIGA agarose gel DNA extraction kit instructions (Tianwei, Nanjing Biological Technology Co., Ltd. DC3511/DC3514) and subcloned into the pMD™ 19-T vector (TaKaRa). The targeted fragments were isolated by sequence analysis of the cloned fragments. The nucleotide sequence of each clone was determined by Shanghai Invitrogen Biotechnology Service Company. The alignment of the flanking sequences of the two kinds of herbicide tolerant transgenic oilseed rape and the chromosome sequences was through NCBI (http://www.ncbi.nlm.nih.gov/BLAST), and the insertion position of foreign gene on chromosome was determined.

Results

Flanking sequences analysis of glyphosate-tolerant and glufosinate-tolerant oilseed rape.

We obtained two flanking sequences about 1250 bp and 600 bp (Figure 1) of glyphosate-tolerant oilseed rape and two flanking sequences about 1600 bp and 590 bp (Figure 4) of glufosinate-tolerant oilseed rape, respectively. The flanking sequences were aligned with

oilseed rape genome sequences and a 181 bp length fragment of GT73/RT73 (Figure 2), a 1920 bp length fragment of T45 (HCN28) (Figure 5) was aligned to the genome sequence, respectively. The matching rate of the flanking sequences of glyphosate-tolerant oilseed rape with A1 chromosome was 100% (Figure 3), and the matching rate of the flanking sequences of glufosinate-tolerant oilseed rape with C8 chromosome 98% (Figure 6). Therefore, we hypothesize that the exogenous *cp4-epsps* gene of glyphosate-tolerant transgenic oilseed rape is located on the A-chromosome, while the exogenous *pat* gene of glufosinate-tolerant transgenic oilseed rape is located on the C-chromosome.

Table 1 Primers used for *cp4-epsps* and *pat* gene flanking sequence amplification.

Primer name	Primer sequence	Application
EPSL-1	5'-GTGGCTGACTTGCGTGTTCGTTCTTCTACT-3'	5 'flanking sequence amplification of *cp4-epsps* gene（1st 1 round）
EPSL-2	5'-ACGGTGTTGATTGCGATGAAGGTGAGAC-3'	5 'flanking sequence amplification of *cp4-epsps* gene（1st 2 round）
EPSL-3	5'-GCTTCCTCGTTATGGGTCTCGTTTCTG-3'	5 'flanking sequence amplification of *cp4-epsps* gene（1st 3 round）
FMVL-1	5'-CCATTCCTTTTTTGTCTGGTCCCCAC -3'	5 'flanking sequence amplification of *FMV 35S* gene（2st 1 round）
FMVL-2	5'-GCTTTTGGCTAATGGTTTGGAGACTC -3'	5 'flanking sequence amplification of *FMV 35S* gene（2st 2 round）
FMVL-3	5'-GAGTTCCTTCTTGGTTTTGGCGATAC -3'	5 'flanking sequence amplification of *FMV 35S* gene（2st 3 round）
PATL-1	5'-TGTGTATCCCAAAGCCTCATGCAACCTAAC-3'	5 'flanking sequence amplification of *pat* gene（1st 1 round）
PATL-2	5'-TAAGCAATACCAGCCACAACACCCTCAACC-3'	5 'flanking sequence amplification of *pat* gene（1st 2 round）
PATL-3	5'-TGTGTATCCCAAAGCCTCATGCAACCTAAC-3'	5 'flanking sequence amplification of *pat* gene（1st 3 round））
CaMV L-1	5'-TCTTGTGGTGTTTGTGGCTCTGTCCTAAAG-3'	5 'flanking sequence amplification of *CaMV 35S* gene（2st 1 round）
CaMV L-2	5'-TTGCGAAGGATAGTGGGATTGTGCGTCAT-3'	5 'flanking sequence amplification of *CaMV 35S* gene（2st 2 round）

CaMV L-3	5'-ACCCTTTGTTGAAAAGTCTCAA TTGCCCTT-3'	5 'flanking sequence amplification of *CaMV 35S* gene（2st 3 round）
M13-F<br>M13-R	5'-CGCCAGGGTTTTCCCAGTCACG AC-3'<br>5'-AGCGGATAACAATTTCACACA GGA-3'	Detection and identification of transformants
ADP1	5'-NGTCGASWGANAWGAA-3'	5 'and 3 'flanking sequence amplification（3 rounds of common）
ADP2	5'-WGTGNAGWANCANAGA-3'	5 'and 3 'flanking sequence amplification（3 rounds of common）
ADP3	5'-AGWGNAGWANCAWAGG-3'	5 'and 3 'flanking sequence amplification（3 rounds of common）
ADP4	5'-GTNCGASWCANAWGTT-3'	5 'and 3 'flanking sequence amplification（3 rounds of common）
ADP5	5'-TCSTNCGNACNTWGGA-3'	5 'and 3 'flanking sequence amplification（3 rounds of common）
ADP6	5'-CAWCGNCNGANASGAA-3'	5 'and 3 'flanking sequence amplification（3 rounds of common）
ADP7	5'- AGTGNAGAANCAAAGG -3'	5 'and 3 'flanking sequence amplification（3 rounds of common）
ADP8	5'- CATCGNCNGANACGAA -3'	5 'and 3 'flanking sequence amplification（3 rounds of common）
ADP9	5'- TGWGNAGWANCASAGA -3'	5 'and 3 'flanking sequence amplification（3 rounds of common）
ADP10	5'- TCGTNCGNACNTAGGA -3'	5 'and 3 'flanking sequence amplification（3 rounds of common）
ADP11	5'- NTCGASTWTSGWGTT -3'	5 'and 3 'flanking sequence amplification（3 rounds of common）
ADP12	5'- STTGNTASTNCTNTGC -3'	5 'and 3 'flanking sequence amplification（3 rounds of common）

Note：ADP: Merging primers

Table 2. The Tail- PCR reaction system of *cp4-epsps* and *pat* gene.

Reaction components	Concentration	Volume (μL)
Template	1.0μg·μL^{-1}	1.0
Gene-specific primers	15.0μM	1.0（1round）1.5（2、3round）
ADP1	15.0μM	2.0（1round）1.0（2、3round）
ADP2	15.0μM	2.0（1round）1.0（2、3round）
ADP3	15.0μM	2.0（1round）1.0（2、3round）
ADP4	15.0μM	2.0（1round）1.0（2、3round）
ADP5	15.0μM	2.0（1round）1.0（2、3round）
ADP6	15.0μM	2.0（1round）1.0（2、3round）
ADP7	15.0μM	2.0（1round）1.0（2、3round）
ADP8	15.0μM	2.0（1round）1.0（2、3round）
ADP9	15.0μM	2.0（1round）1.0（2、3round）
ADP10	15.0μM	2.0（1round）1.0（2、3round）
ADP11	15.0μM	2.0（1round）1.0（2、3round）
ADP12	15.0μM	2.0（1round）1.0（2、3round）
ddH_2O	-	14.1（1round）14.6（2、3round）
Total Volume	-	25.0

Note：ADP: Merging primers

Table 3 The Tail-PCR programmes of *cp4-epsps* and *pat* gene.

Reaction	Program No.	Number of cycles	Cycle parameters
1round	1	1	94℃ 3 min, 95℃ 2 min
	2	5	94℃ 1 min, 63℃ 1 min, 72℃ 3 min
	3	1	94℃ 30 s, 41 ℃ 3 min to 0.2℃/s to rise to 72℃, 72℃ 3 min
	4	15	94℃ 30 s, 63 ℃ 1 min, 72℃ 2 min;<br>94℃ 30 s, 63 ℃ 1 min, 72℃ 2 min;<br>94℃ 30 s, 41 ℃ 1 min, 72℃ 2 min
2round	5	1	72℃ 10 min
	1	1	94℃ 3 min, 95℃ 2 min
	6	12	94℃ 30 s, 63 ℃ 1 min, 72℃ 2 min;<br>94℃ 30 s, 63 ℃ 1 min, 72℃ 2 min;<br>94℃ 30 s, 41 ℃ 1 min, 72℃ 2 min
3round	5	1	72℃ 10 min
	7	1	94℃ 3 min
	8	15	94℃ 30 s, 63 ℃ 1 min, 72℃ 2 min;<br>94℃ 30 s, 63 ℃ 1 min, 72℃ 2 min;<br>94℃ 30 s, 41 ℃ 1 min, 72℃ 2 min
	5	1	72℃ 10 min

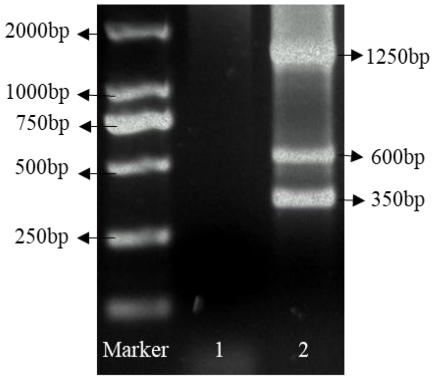

Marker：DL2000;1, 2 refers to the binding primers ADP1 and ADP2

Fig.1 The results for third round Tail-PCR amplification results of glyphosate-tolerant transgenic oilseed rape promoter (FMV35S) left flanking sequences

	Description	Max score	Total score	Query cover	E value	Ident	Accession
☑	Sequence 8 from patent US 7718373	275	275	100%	2e-71	100%	GX257885.1

```
>GX257885.1:166-322 Sequence 8 from patent US 7718373
TTACTCTTTCTTTTTCTCCATATTGACCATCATACTCATTGCTGATCCATGTAGATTTCCCGGACATGAA
GATCATCCTCCTTCCTTTCCTTGCCTTTCCTTCCTTTTCTTGCCTTCGTATAAGCTTGTGTCAATTGTTG
ACAGAGAATCTTGCTGA
```

Figure 2 Comparison of left border of glyphosate-tolerant transgenic oilseed rape transformant in Blast of NCBI

```
>NC_027757.1 Brassica napus cultivar ZS11 chromosome A1,
                Brassica_napus_assembly_v1.0, whole genome shotgun sequence
          Length = 46747018

 Score =  119 bits (60), Expect = 1e-025
 Identities = 60/60 (100%)
 Strand = Plus / Minus


Query: 93       tccttccttttcttgccttcgtataagcttgtgtcaattgttgacagagaatcttgctga 152
                ||||||||||||||||||||||||||||||||||||||||||||||||||||||||||||
Sbjct: 41358494 tccttccttttcttgccttcgtataagcttgtgtcaattgttgacagagaatcttgctga 41358435
```

Figure 3 Comparison of the left border of the glyphosate-tolerant transgenic oilseed rape transformants with the oilseed rape whole genome sequence

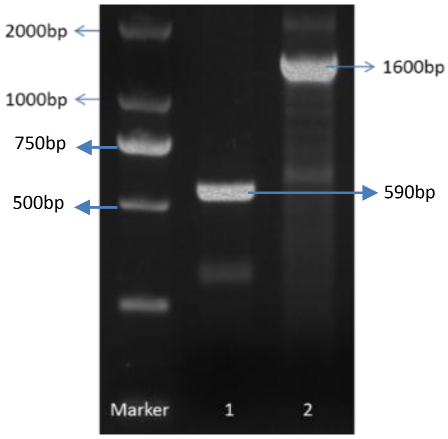

Marker： DL2000; 1, 2： the binding primers ADP1 and ADP2

Figure 4 The results for third round Tail-PCR amplification results of glufosinate-tolerant transgenic oilseed rape promoter (CaMV35S) left flanking sequence

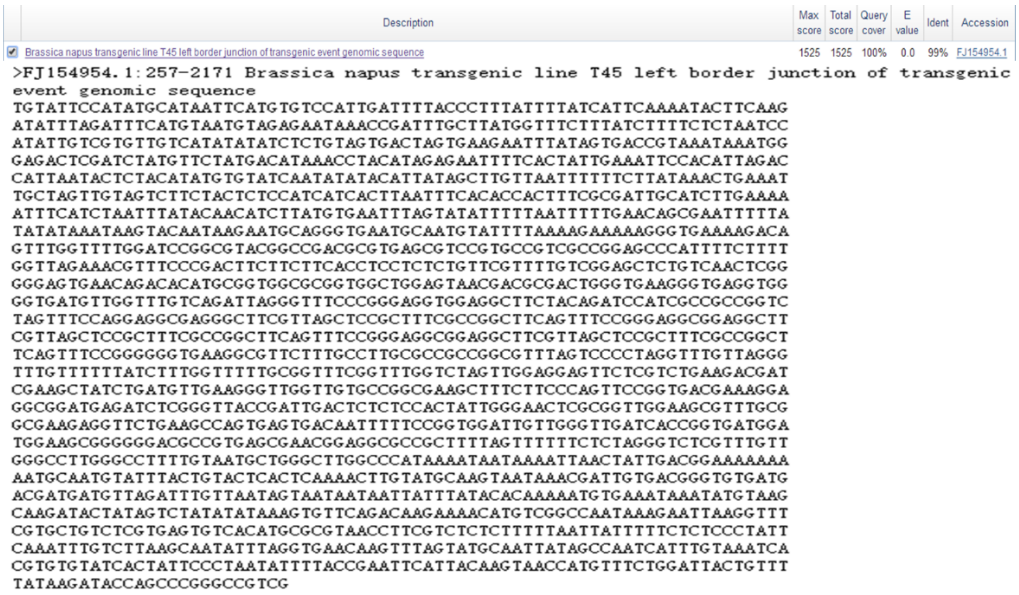

Description	Max score	Total score	Query cover	E value	Ident	Accession
Brassica napus transgenic line T45 left border junction of transgenic event genomic sequence	1525	1525	100%	0.0	99%	FJ154954.1

>FJ154954.1:257-2171 Brassica napus transgenic line T45 left border junction of transgenic event genomic sequence

TGTATTCCATATGCATAATTCATGTGTCCATTGATTTTACCCTTTATTTTATCATTCAAAATACTTCAAG
ATATTTAGATTTCATGTAATGTAGAGAATAAACCGATTTGCTTATGGTTTCTTTATCTTTTCTCTAATCC
ATATTGTCGTGTTGTCATATATATCTCTGTAGTGACTAGTGAAGAATTTATAGTGACCGTAAATAAATGG
GAGACTCGATCTATGTTCTATGACATAAACCTACATAGAGAATTTTCACTATTGAAATTCCACATTAGAC
CATTAATACTCTACATATGTGTATCAATATATACATTATAGCTTGTTAATTTTTTCTTATAAACTGAAAT
TGCTAGTTGTAGTCTTCTACTCTCCATCATCACTTAATTTCACACCACTTTCGCGATTGCATCTTGAAAA
ATTTCATCTAATTTATACAACATCTTATGTGAATTTAGTATATTTTTAATTTTTGAACAGCGAATTTTTA
TATATAAATAAGTACAATAAGAATGCAGGGTGAATGCAATGTATTTTAAAAGAAAAAGGGTGAAAAGACA
GTTTGGTTTTGGATCCGGCGTACGGCCGACGCGTGAGCGTCCGTGCCGTCGCCGGAGCCCATTTTCTTTT
GGTTAGAAACGTTTCCCGACTTCTTCTTCACCTCCTCTCTGTTCGTTTTGTCGGAGCTCTGTCAACTCGG
GGGAGTGAACAGACACATGCGGTGGCGCGGTGGCTGGAGTAACGACGCGACTGGGTGAAGGGTGAGGTGG
GGTGATGTTGGTTTGTCAGATTAGGGTTTCCCGGGAGGTGGAGGCTTCTACAGATCCATCGCCGCCGGTC
TAGTTTCCAGGAGGCGAGGGCTTCGTTAGCTCCGCTTTCGCCGGCTTCAGTTTCCGGGAGGCGGAGGCTT
CGTTAGCTCCGCTTTCGCCGGCTTCAGTTTCCGGGAGGCGGAGGCTTCGTTAGCTCCGCTTTCGCCGGCT
TCAGTTTCCGGGGGGTGAAGGCGTTCTTTGCCTTGCGCCGCCGGCGTTTAGTCCCCTAGGTTTGTTAGGG
TTTGTTTTTTATCTTTGGTTTTTGCGGTTTCGGTTTGGTCTAGTTGGAGGAGTTCTCGTCTGAAGACGAT
CGAAGCTATCTGATGTTGAAGGGTTGGTTGTGCCGGCGAAGCTTTCTTCCCAGTTCCGGTGACGAAAGGA
GGCGGATGAGATCTCGGGTTACCGATTGACTCTCTCCACTATTGGGAACTCGCGGTTGGAAGCGTTTGCG
GCGAAGAGGTTCTGAAGCCAGTGAGTGACAATTTTTCCGGTGGATTGTTGGGTTGATCACCGGTGATGGA
TGGAAGCGGGGGGACGCCGTGAGCGAACGGAGGCGCCGCTTTTAGTTTTTTCTCTAGGGTCTCGTTTGTT
GGGCCTTGGGCCTTTTGTAATGCTGGGCTTGGCCCATAAAATAATAAAATTAACTATTGACGGAAAAAAA
AATGCAATGTATTTACTGTACTCACTCAAAACTTGTATGCAAGTAATAAACGATTGTGACGGGTGTGATG
ACGATGATGTTAGATTTGTTAATAGTAATAATAATTATTTATACACAAAAATGTGAAATAAATATGTAAG
CAAGATACTATAGTCTATATATAAAGTGTTCAGACAAGAAAACATGTCGGCCAATAAAGAATTAAGGTTT
CGTGCTGTCTCGTGAGTGTCACATGCGCGTAACCTTCGTCTCTCTTTTTAATTATTTTTCTCTCCCTATT
CAAATTTGTCTTAAGCAATATTTAGGTGAACAAGTTTAGTATGCAATTATAGCCAATCATTTGTAAATCA
CGTGTGTATCACTATTCCCTAATATTTTACCGAATTCATTACAAGTAACCATGTTTCTGGATTACTGTTT
TATAAGATACCAGCCCGGGCCGTCG

Figure 5 Comparison of the left border of the glufosinate-tolerant transgenic oilseed rape transformant in Blast of NCBI

```
>NC_027774.1 Brassica napus cultivar ZS11 chromosome C8,
                Brassica_napus_assembly_v1.0, whole genome shotgun sequence
          Length = 36757632

 Score = 1675 bits (845), Expect = 0.0
 Identities = 882/892 (98%), Gaps = 3/892 (0%)
 Strand = Plus / Minus


Query: 9        atatgcataattcatgtgtccattgattttaccctttattttatcattcaaaatacttca 68
                 ||||||||||||||||||||||||||||||||||||||||||||||||||||||||||||
Sbjct: 35552695 atatgcataattcatgtgtccattgattttaccctttattttatcattcaaaatacttca 35552636


Query: 69       agatatttagatttcatgtaatgtagagaataaaccgatttgcttatggtttctttatct 128
                 ||||||||||||||||||||||||||||||||||||||||||||||||||||||||||||
Sbjct: 35552635 agatatttagatttcatgtaatgtagagaataaaccgatttgcttatggtttctttatct 35552576


Query: 129      tttctctaatccatattgtcgtgttgtcatatatatctctgtagtgactagtgaagaatt 188
                 ||||||||||||||||||||||||||||||||||||||||||||||||||||||||||||
Sbjct: 35552575 tttctctaatccatattgtcgtgttgtcatatatatctctgtagtgactagtgaagaatt 35552516


Query: 189      tatagtgaccgtaaataaatgggagactcgatctatgttctatgacataaacctacatag 248
                 ||||||||||||||||||||||||||||||||||||||||||||||||||||||||||||
Sbjct: 35552515 tatagtgaccgtaaataaatgggagactcgatctatgttctatgacataaacctacatag 35552456


Query: 249      agaattttcactattgaaattccacattagaccattaatactctacatatgtgtatcaat 308
                 |||||||||||||||||||||||||||||||||||||||||||||||||||||||||| |
Sbjct: 35552455 agaattttcactattgaaattccacattagaccattaatactctacatatgtgtatcagt 35552396


Query: 489      tatatataaataagtacaataagaatgcagggtgaatgcaatgtattttaaaagaaaaag 548
                 |||||||||||||||||||||||||||||||||||| ||||||||||| |||||||||||
Sbjct: 35552215 tatatataaataagtacaataagaatgcagggtgaacgcaatgtatttaaaaagaaaaag 35552156


Query: 549      ggtgaaaagacagtttggttttggatccggcgtacggccgacgcgtgagcgtccgtgccg 608
                 ||||||||||||||||||||||||||||||||||||||||||||||||||||||||||||
Sbjct: 35552155 ggtgaaaagacagtttggttttggatccggcgtacggccgacgcgtgagcgtccgtgccg 35552096


Query: 609      tcgccggagcccattttcttttggttagaaacgtttcccgacttcttcttcacct--cct 666
                 ||||||||||||||||||||||||||||||||||| |||||||||||||||||||   ||
Sbjct: 35552095 tcgccggagcccattttcttttggttagaaacgttccccgacttcttcttcaccttctct 35552036


Query: 667      ctctgttcgttttgtcggagctctgtcaactcggggagtgaacagacacatgcggtggc 726
                 |||||||||||||||||||||||||||||||| |||||||||||||||||||||||||||
Sbjct: 35552035 ctctgttcgttttgtcggagctctgtcaactc-ggggagtgaacagacacatgcggtggc 35551977


Query: 727      gcggtggctggagtaacgacgcgactgggtgaagggtgaggtggggtgatgttggtttgt 786
                 ||||||||||||||||||||||||||||||||||||||||||||||||||||||||||||
Sbjct: 35551976 gcggtggctggagtaacgacgcgactgggtgaagggtgaggtggggtgatgttggtttgt 35551917


Query: 787      cagattagggtttcccgggaggtggaggcttctacagatccatcgccgccggtctagttt 846
                 |||||||||||||||||||||||||||| |||||||||||||||||||||||||||||||
Sbjct: 35551916 cagattagggtttcccgggaggtggaggtttctacagatccatcgccgccggtctagttt 35551857


Query: 847      ccaggaggcgagggcttcgttagctccgctttcgccggcttcagtttccggg 898
                 || |||||||||||||||||||||||||||||||||||||||||||||||||
Sbjct: 35551856 ccgggaggcgagggcttcgttagctccgctttcgccggcttcagtttccggg 35551805
```

Figure 6 Comparison of the left border of the glufosinate-tolerant transgenic oilseed rape transformants with the oilseed rape whole genome sequence

Reference:

Liu YG, Whittier RF (1995) Thermal asymmetric interlaced PCR: automatable amplification and sequencing of insert end fragments from P1 and YAC clones for chromosome walking. Genomics, 25, 674–681.
